# HPV prevalence in esophageal cancer: an updated systematic review and meta-analysis

**DOI:** 10.3389/fmicb.2026.1771173

**Published:** 2026-04-07

**Authors:** Yi Nan, Jiao Su, Jingjing Li, Yuanhao Liang, Xiaofeng He

**Affiliations:** 1 Department of Gastrointestinal Surgery, Heping Hospital Affiliated to Changzhi Medical College, Changzhi, China; 2 Department of Biochemistry, Changzhi Medical College, Changzhi, China; 3 Institute of Evidence-Based Medicine, Heping Hospital Affiliated to Changzhi Medical College, Changzhi, China; 4 School of Public Health, Southern Medical University, Guangzhou, China

**Keywords:** esophageal cancer, human papillomavirus, meta-analysis, odds ratios, prevalence

## Abstract

**Objectives:**

Esophageal cancer (EC) is a significant global health concern. Human papillomavirus (HPV) has been proposed as a potential etiological factor, though its role, especially in esophageal adenocarcinoma (EAC), remains controversial. This study aims to systematically review and update the meta-analysis of HPV prevalence in both esophageal squamous cell carcinoma (ESCC) and EAC, and to assess the association between HPV infection and EC risk.

**Methods:**

A comprehensive literature search of PubMed, Scopus, and Web of Science was performed to identify studies published between January 1, 2000, and November 28, 2025 that reported HPV prevalence in EC, with a specific focus on both ESCC and EAC. Data on HPV genotypes, detection methods, and study characteristics were extracted and analyzed using random-effects models. Prevalence estimates were calculated for various detection methods, and meta-regression was used to identify sources of heterogeneity.

**Results:**

A total of 151 studies, including 18,913 EC cases, were analyzed. HPV prevalence in EC varied by detection method, with polymerase chain reaction (PCR)-based studies showing an overall prevalence of 31% (95% CI: 25%−36%). Regionally, East Asia exhibited the highest prevalence (44%, 95% CI: 35%−53%). HPV prevalence was consistently higher in ESCC (29%, 95% CI: 23%−36%) compared to EAC (16%, 95% CI: 5%−30%). HPV-16 was the most prevalent genotype, showing a stronger association with ESCC than EAC. Meta-regression identified geographic region as a significant predictor of HPV prevalence. A pooled odds ratio (OR) of 2.92 (95% CI: 2.19–3.90) indicates a strong association between HPV infection and increased EC risk.

**Conclusions:**

HPV infection—particularly HPV-16—is epidemiologically associated with esophageal cancer, with higher detection rates observed in ESCC. However, current evidence is insufficient to establish HPV as a definitive causal driver of EC, especially for EAC, due to the predominance of observational designs and limited mechanistic confirmation.

## 1 Introduction

Esophageal cancer (EC) ranks as the seventh most common malignancy and the sixth leading cause of cancer-related mortality worldwide, accounting for an estimated 576,529 new cases and 538,602 deaths in 2021 (Jin et al., 2025). Characterized by rapid progression and poor prognosis, EC presents primarily as esophageal squamous cell carcinoma (ESCC) in high-incidence regions such as East Asia and sub-Saharan Africa, whereas esophageal adenocarcinoma (EAC) predominates in Western countries (Morgan et al., 2022). Although tobacco use, alcohol consumption, and dietary deficiencies are well-established risk factors, the etiology of EC is multifactorial, with increasing attention directed toward infectious agents such as human papillomavirus (HPV) (Lander et al., 2023; Jiang et al., 2025).

HPV, a double-stranded DNA virus comprising more than 200 genotypes, is a recognized carcinogen in anogenital and oropharyngeal cancers (Han et al., 2024). High-risk types, particularly HPV-16 and HPV-18, promote carcinogenesis through E6 and E7 oncoproteins that inactivate the tumor suppressors p53 and Rb (Zhang et al., 2025). Interest in HPV as a potential contributor to EC dates back to the 1980s (Syrjänen, 2002). HPV infection likely promote malignant transformation of esophageal epithelium, but epidemiological and mechanistic findings remain inconsistent, even in high-incidence regions (Guo et al., 2012; Halec et al., 2016). This inconsistency is partly due to variations in HPV detection methods, population characteristics, and confounders such as smoking and alcohol use (Petrick et al., 2014). Moreover, prior meta-analyses have predominantly focused on ESCC, leaving the role of HPV in EAC largely unexplored (Petrick et al., 2014; Hussain et al., 2022). Given the rising incidence of EAC in many Western populations (Coleman et al., 2018), the absence of robust pooled estimates of HPV prevalence in EAC presents a critical gap in the literature (Dhesy-Thind, 2018).

Therefore, this systematic review and updated meta-analysis was undertaken not only to provide an updated estimate of HPV prevalence in esophageal cancer, but also to address several specific and previously unresolved questions. We aimed to determine whether HPV prevalence differs between ESCC and EAC; whether high-risk HPV genotypes, particularly HPV-16 and HPV-18, show distinct prevalence patterns across histological subtypes; and whether HPV infection is consistently associated with an increased risk of esophageal cancer. In addition, we sought to explore the extent to which geographic region and methodological factors, including HPV detection approaches, contribute to the substantial heterogeneity observed across studies.

## 2 Methods

### 2.1 Search strategy and selection criteria

A comprehensive systematic literature search was conducted to identify studies reporting the prevalence of HPV infection in patients with EC, with a focus on both ESCC and EAC. Electronic databases—PubMed, Scopus, and Web of Science—were searched for articles published between January 1, 2000, and November 28, 2025. The search strategy combined keywords and Medical Subject Headings (MeSH) terms related to HPV, EC, and their histological subtypes. Key search terms included “human papillomavirus,” “HPV,” “esophageal cancer,” “esophageal squamous cell carcinoma,” “esophageal adenocarcinoma,” and “HPV prevalence.” The full search string was: (“human papillomavirus” OR “HPV” OR “papillomavirus”) AND (“esophageal cancer” OR “esophageal carcinoma” OR “esophageal neoplasm” OR “esophagus cancer” OR “esophageal cancer”).

This review followed the Preferred Reporting Items for Systematic Reviews and Meta-Analyses (PRISMA) 2020 guidelines (Page et al., 2021). Eligibility criteria were defined using the PICOS framework:

(1) Population (P): adults diagnosed with EC (ESCC, EAC, or other types);
(2) Intervention (I): not applicable for observational designs;
(3) Comparison (C): individuals without EC, without HPV-related cancers, or without esophageal pathology;
(4) Outcome (O): HPV prevalence in EC or the association between HPV and EC risk;
(5) Study design (S): observational cohort, case-control, or cross-sectional studies reporting subtype-specific HPV prevalence or risk estimates.

Only English-language original research articles were included. We excluded reviews, conference abstracts, opinion pieces, letters, case reports, case series, and duplicate datasets; when multiple publications drew from the same cohort, the most complete or most recent report was retained.

### 2.2 Data extraction

Literature screening was performed using EndNote X9. Two authors (YN and JS) independently extracted data using a standardized form. Extracted variables included the first author, publication year, study country, HPV sample source, HPV genotypes, detection methods, PCR primers or serology antibodies, EC histological subtype, sample size, and HPV prevalence for ESCC and EAC. When provided, effect estimates (risk ratios or odds ratios with 95% confidence intervals) were also collected. Study quality and risk of bias were assessed using the Newcastle–Ottawa Scale (NOS). Studies scoring >7 points were considered high quality, scores of 5–7 moderate quality, and ≤ 4 low quality. Discrepancies were resolved by discussion or, when necessary, consultation with a third author (XH) to ensure accuracy and completeness.

Screening was conducted in three steps. First, duplicates were removed through automated and manual checks. Second, titles and abstracts were screened to exclude clearly irrelevant studies or those not meeting PICOS criteria; records lacking abstracts or containing ambiguous information were retained for full-text review. Third, two authors independently assessed full texts for final eligibility and resolved disagreements through discussion or adjudication. This process ensured consistent application of inclusion criteria and minimized selection bias.

### 2.3 Statistical analysis

To reduce the influence of studies with very low event counts, prevalence data were stabilized using the Freeman–Tukey double arcsine transformation before pooling (Murray and John, 1950). The Wilson method was used to compute 95% confidence intervals to avoid boundary issues associated with asymptotic methods (Wilson, 1927). Pooled odds ratios (ORs) and 95% CIs assessing the association between HPV and EC were calculated using a random-effects model or fixed-effect model according to inter-studies heterogeneity. Heterogeneity was evaluated using Cochran's *Q* (reported as χ^2^ and *p*-value) and the *I*^2^ statistic; *p* < 0.05 or *I*^2^ > 75% indicated substantial heterogeneity (Higgins and Thompson, 2002; Higgins et al., 2003). Given the substantial clinical and methodological diversity across studies (including geographic region, detection techniques, and tumor subtype), we primarily applied random-effects models, assuming that the true HPV prevalence and risk estimates may vary between study settings. Fixed-effect models were used only in analyses where studies were sufficiently homogeneous and where a common underlying effect size could reasonably be assumed. Potential sources of heterogeneity—such as detection method, histological subtypes, region, and study period—were explored using meta-regression. Publication bias was evaluated using funnel plots and Egger's test, with the trim-and-fill method applied when necessary (Begg and Mazumdar, 1994; Egger et al., 1997). All analyses were performed in R software (version 4.2.2; R Foundation for Statistical Computing, Vienna, Austria) using the “metafor” package. Statistical tests were two-sided, with *p* < 0.05 considered significant.

## 3 Results

### 3.1 Study selection and characteristics

**Figure 1:**
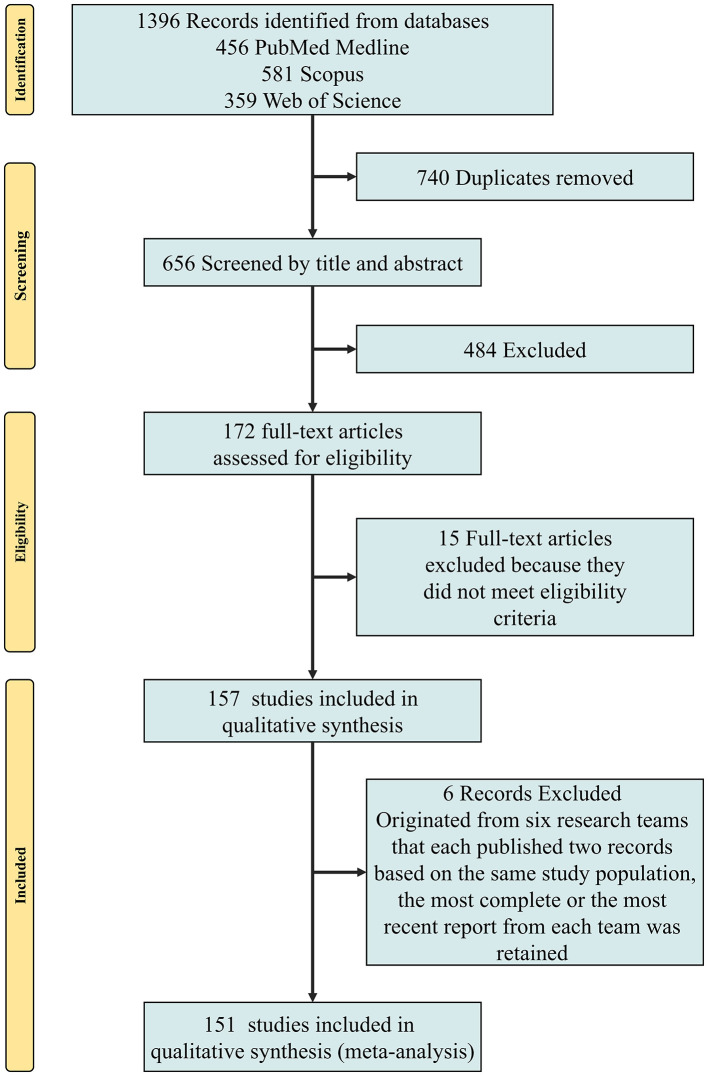
Flow-chart documenting the systematic search conducted to identify included studies.

A systematic search across PubMed, Scopus, and Web of Science initially identified 1,396 records. After removing 740 duplicates and screening 484 records based on titles and abstracts, 172 full-text articles were assessed for eligibility. Fifteen articles were excluded for not meeting inclusion criteria, and six were removed due to overlapping study populations. Ultimately, 151 studies were included in the qualitative synthesis and meta-analysis (Figure 1). The characteristics of these studies are summarized in Supplementary Table S1 (Guo et al., 2012; Reynders et al., 2025; de Oliveira et al., 2025; Qu et al., 2024; Ndemela et al., 2024; Kotian et al., 2024; Yuan et al., 2023; Maroga et al., 2023; Burassakarn et al., 2023; Sadeghian et al., 2022; Munari et al., 2022; Zhang et al., 2021; Woellner et al., 2021; Ishida et al., 2021; Zheng et al., 2020; Zhang et al., 2020; Sultana et al., 2020; Singh et al., 2020; Kanaan et al., 2020; Inoue et al., 2020; Dinc et al., 2020; Tasneem et al., 2019; Parameshwaran et al., 2019; Leon et al., 2019; Baş et al., 2019; Vošmik et al., 2018; Li et al., 2018; Ghaffar et al., 2018; Geßner et al., 2018; Cheah et al., 2018; Zhang et al., 2017; Rajendra et al., 2017; Pastrez et al., 2017; da Costa et al., 2017; Chan et al., 2017; Yahyapour et al., 2016; Soheili et al., 2016; Prakash Saxena et al., 2016; Antonsson et al., 2016; Zou et al., 2015; Wang et al., 2015; Mehryar et al., 2015; Kumar et al., 2015; Kayamba et al., 2015; Georgantis et al., 2015; Dong et al., 2015; Zhang et al., 2014; Yu et al., 2014; Yang et al., 2014; Teng et al., 2014; Ludmir et al., 2014; Liu et al., 2014; He et al., 2014; Cui et al., 2014; Chen et al., 2014; Cao et al., 2014; Yang et al., 2013; Yahyapour et al., 2013; Wang et al., 2013; Vaiphei et al., 2013; Schäfer et al., 2013; Rajendra et al., 2013; Qi et al., 2013; Mohiuddin et al., 2013; Liu et al., 2013; Hu J. M. et al., 2013; Hu et al., 2013; Haeri et al., 2013; Feng et al., 2013; Antunes et al., 2013; Qu et al., 2012; Noori et al., 2012; Löfdahl et al., 2012; Hu et al., 2012; Herbster et al., 2012; Gupta et al., 2012; Dabrowski et al., 2012; Abdirad et al., 2012; Zhang Q. Y. et al., 2011; Zhang D. H. et al., 2011; Malik et al., 2011; Iyer et al., 2011; Hussain et al., 2011; Goto et al., 2011; Cui et al., 2011; Castillo et al., 2011; Ayshamgul et al., 2011; Zhang et al., 2010; Wang et al., 2010; Liu et al., 2010; Koshiol et al., 2010; Antonsson et al., 2010; Zhao et al., 2009; Tornesello et al., 2009; Liao et al., 2009; Herrera-Goepfert et al., 2009; Bellizzi et al., 2009; Yang et al., 2008; Lyronis et al., 2008; Lu et al., 2008; Li et al., 2008; Koh et al., 2008; Bognar et al., 2008; Zhou et al., 2007; Shuyama et al., 2007; Pantelis et al., 2007; Mir et al., 2007; Matsha et al., 2007; Liu et al., 2007; Far et al., 2007; Dai et al., 2007; Yao et al., 2006; Souto Damin et al., 2006; Qi et al., 2006; Kamangar et al., 2006; Dreilich et al., 2006; Castillo et al., 2006; Zhu et al., 2005; White et al., 2005; Lyronis et al., 2005; Liu et al., 2005; Katiyar et al., 2005; Farhadi et al., 2005; Cao et al., 2005; Bahnassy et al., 2005; Xu et al., 2004; Si et al., 2004; Lu et al., 2004; Acevedo-Nuño et al., 2004; Zhou et al., 2003; Xu et al., 2003; Weston and Prolla, 2003; Van Doornum et al., 2003; Si et al., 2003; Liu et al., 2003; Awerkiew et al., 2003; Shen et al., 2002; Matsha et al., 2002; Li et al., 2002; Hasegawa et al., 2002; Sobti et al., 2001; Peixoto Guimaraes et al., 2001; Lu et al., 2001; Astori et al., 2001; Tripodi et al., 2000; Talamini et al., 2000; Lambot et al., 2000; Kawaguchi et al., 2000; Kamath et al., 2000; Chang et al., 2000a,b). Study sample sizes ranged from 17 to 1,435 EC cases (median = 74, interquartile range: 45, 150), with a total of 18,913 EC cases included across all studies. Study quality was assessed using the NOS (Supplementary Tables S2, S3), with most studies rated as having low to moderate risk of bias. While selection and outcome/exposure assessment were generally adequate, comparability was frequently limited due to insufficient control for key confounders. These methodological constraints likely contributed to the substantial between-study heterogeneity observed in the pooled analyses.

### 3.2 Prevalence of HPV infection in esophageal cancer

The overall prevalence of HPV infection in EC was analyzed using various detection methods. PCR-based studies reported an HPV prevalence of 31% (95% CI: 25%−36%), with significant heterogeneity (*I*^2^ = 97.8%, *p* < 0.0001). In PCR-based analyses stratified by primer type (Supplementary Table S4), pooled HPV prevalence ranged from 30 to 49% across different primer systems. Consensus primers MY09/11 and GP5+/6+ showed similar prevalence estimates (34 and 35%, respectively), while SPF10-based assays reported higher prevalence (49%) with wide confidence intervals. Type-specific PCR assays yielded a pooled prevalence of 31% and demonstrated lower heterogeneity compared with consensus primer systems. However, high heterogeneity persisted across most primer categories, suggesting that primer selection alone does not fully explain between-study variability. Similar pooled prevalences were observed using *in situ* hybridization (ISH) (26%, 95% CI: 13%−41%), immunohistochemistry (IHC) (41%, 95% CI: 27%−55%), enzyme-linked immunosorbent assay (40%, 95% CI: 17%−66%), and other methods (31%, 95% CI: 7%−62%). However, heterogeneity remained high across methods (Table 1).

**Table 1:** Random-effects meta-analysis of HPV prevalence in esophageal cancer stratified by detection method and study characteristics.

Characteristic	Studies (*N)*	EC patients (*N*)	HPV positive (*N*)	Prevalence (95% CI)	Heterogeneity (Random effects model)	
					*I* ^2^	***p*** **-Value**
Polymerase chain reaction						
Overall	118	13,535	4,797	0.31 (0.25, 0.36)	97.8%	< 0.0001
Histological subtypes						
ESCC	90	11,261	4,106	0.29 (0.23, 0.36)	98.0%	< 0.0001
EAC	15	841	135	0.16 (0.05, 0.30)	94.0%	< 0.0001
Region						
Europe	16	1,222	222	0.18 (0.09, 0.30)	90.0%	< 0.0001
Africa	10	666	201	0.21 (0.03, 0.47)	97.1%	< 0.0001
South Asia	10	668	187	0.30 (0.12, 0.52)	93.2%	< 0.0001
East Asia	54	8,370	3,711	0.44 (0.35, 0.53)	98.3%	< 0.0001
Middle East and North Africa	12	912	191	0.19 (0.07, 0.34)	93.5%	< 0.0001
South America	8	827	133	0.15 (0.07, 0.26)	83.4%	< 0.0001
Oceania	5	556	55	0.18 (0.00, 0.60)	97.0%	< 0.0001
Southeast Asia	1	67	1	0.01 (0.00, 0.09)	/	/
North America	5	247	96	0.38 (0.03, 0.84)	95.5%	< 0.0001
Study period						
2000–2009	50	4,655	1,599	0.32 (0.24, 0.41)	96.9%	< 0.0001
2010–2019	56	6,538	2,205	0.28 (0.20, 0.37)	98.3%	< 0.0001
2020–present	12	2,342	993	0.39 (0.24, 0.56)	97.1%	< 0.0001
HPV genotype						
HPV-16	74	8,877	2,485	0.25 (0.20, 0.30)	95.8%	< 0.0001
HPV-18	42	5,863	801	0.09 (0.02, 0.20)	95.2%	< 0.0001
*In situ* hybridization						
Overall	14	2,509	576	0.26 (0.13, 0.41)	96.5%	< 0.0001
Histological subtypes						
ESCC	13	2,328	561	0.27 (0.13, 0.44)	96.5%	< 0.0001
Region						
East Asia	12	2,461	575	0.31 (0.17, 0.46)	96.9%	< 0.0001
North America	2	48	1	0.01 (0.00, 0.95)	35.6%	0.2128
Study period						
2000–2009	9	1,881	429	0.30 (0.11, 0.54)	97.0%	< 0.0001
2010–2019	3	305	45	0.13 (0.00, 0.49)	89.5%	< 0.0001
2020–present	2	323	102	0.27 (0.00, 1.00)	98.4%	< 0.0001
HPV genotype						
HPV-16	7	1,293	272	0.34 (0.08, 0.65)	98.9%	< 0.0001
HPV-18	2	803	14	0.02 (0.00, 0.05)	0%	0.6966
Immunohistochemistry						
Overall	13	768	265	0.41 (0.27, 0.55)	89.0%	< 0.0001
Histological subtypes						
ESCC	12	717	235	0.39 (0.23, 0.57)	91.8%	< 0.0001
EAC	1	36	15	0.42 (0.20, 0.62)	/	/
					*I* ^2^	***p*** **-Value**
Region						
Europe	1	86	18	0.21 (0.13, 0.31)	/	/
South Asia	2	131	36	0.32 (0.00, 1.00)	84.7%	< 0.0001
East Asia	7	432	148	0.39 (0.17, 0.64)	90.0%	< 0.0001
South America	1	58	27	0.47 (0.33, 0.60)	/	/
North America	2	61	36	0.64 (0.00, 1.00)	91.3%	< 0.0001
Study period						
2000–2009	4	208	114	0.38 (0.00, 0.89)	91.1%	< 0.0001
2010–2019	4	216	90	0.51 (0.11, 0.90)	93.3%	< 0.0001
2020–present	5	344	21	0.35 (0.19, 0.53)	82.4%	0.0001
HPV genotype						
HPV-16	6	309	124	0.46 (0.20, 0.73)	90.0%	< 0.0001
Enzyme-linked immunosorbent assay						
Overall	9	3,204	2,081	0.40 (0.17, 0.66)	99.6%	< 0.0001
Histological subtypes						
ESCC	8	3,043	2,066	0.46 (0.22, 0.72)	99.5%	< 0.0001
EAC	1	48	11	0.23 (0.11, 0.37)	/	/
Region						
Europe	1	117	20	0.17 (0.11, 0.25)	/	/
East Asia	8	3,087	2,061	0.44 (0.17, 0.72)	99.6%	< 0.0001
Study period						
2000–2009	3	316	56	0.16 (0.00, 0.66)	94.6%	< 0.0001
2010–2019	6	2,888	2,025	0.54 (0.23, 0.84)	99.6%	< 0.0001
HPV genotype						
HPV-16	10	3,083	788	0.24 (0.12, 0.40)	98.4%	< 0.0001
HPV-18	2	1,534	83	0.06 (0.00, 0.30)	35.2%	0.2143
Other						
Overall	8	609	208	0.31 (0.07, 0.62)	97.7%	< 0.0001
Histological subtypes						
ESCC	7	544	197	0.38 (0.11, 0.70)	97.7%	< 0.0001
EAC	2	65	11	0.13 (0.00, 1.00)	95.7%	< 0.0001
Region						
Europe	1	37	0	0.00 (0.00, 0.09)	/	/
South Asia	1	18	9	0.50 (0.26, 0.74)	/	/
East Asia	4	414	155	0.45 (0.01, 0.96)	98.7%	< 0.0001
South America	1	40	1	0.02 (0.00, 0.13)	/	/
Southeast Asia	1	100	43	0.43 (0.33, 0.53)	/	/
Study period						
2000–2009	1	40	1	0.02 (0.00, 0.14)	/	/
2010–2019	6	469	164	0.35 (0.04, 0.77)	98.1%	< 0.0001
2020–present	1	100	43	0.43 (0.33, 0.53)	/	/
					*I* ^2^	***p*** **-Value**
HPV genotype						
HPV-16	5	444		0.27 (0.01, 0.69)	98.0%	< 0.0001
HPV-18	3	178	21	0.12 (0.00, 0.34)	60.8%	0.0779

Among studies assessing E6/E7 mRNA positivity (six studies; *n* = 387), the pooled HPV prevalence was 30% (95% CI: 0–85%), with substantial heterogeneity (*I*^2^ = 98.2%, *p* < 0.0001; Supplementary Figure S1). In studies evaluating viral integration (four studies; *n* = 481), the pooled prevalence was higher at 61% (95% CI: 19%−95%), also with marked heterogeneity (*I*^2^ = 97.2%, *p* < 0.0001). Overall, when combining both biologically informative markers, the pooled prevalence of active or integrated HPV infection was 42% (95% CI: 13%−75%).

There was marked regional variation in HPV prevalence. Among PCR studies, East Asia had the highest prevalence (44%, 95% CI: 35%−53%), followed by South Asia (30%, 95% CI: 12%−52%) and Africa (21%, 95% CI: 3%−47%). In contrast, lower prevalence was observed in Europe (18%, 95% CI: 9%−30%) and South America (15%, 95% CI: 7%−26%). Prevalence estimates from North America and Oceania showed wide confidence intervals, reflecting limited study numbers and substantial heterogeneity (Table 1). To better visualize geographic disparities in HPV prevalence, we generated a world map heat plot illustrating pooled PCR-based HPV prevalence across major regions (Supplementary Figure S2). Over time, HPV prevalence remained stable across study periods, with pooled estimates of 32% (2000–2009), 28% (2010–2019), and 39% (2020–present) for PCR-based analyses (Table 1). This stability suggests that methodological and population differences play a larger role in variability than temporal trends alone.

### 3.3 HPV prevalence by histological subtype and HPV genotype

HPV prevalence was consistently higher in ESCC than in EAC across detection methods (Table 1). PCR studies showed a pooled HPV prevalence of 29% (95% CI: 23%−36%) in ESCC, compared to 16% (95% CI: 5%−30%) in EAC. Similar patterns were observed with immunohistochemistry and ELISA, although EAC-specific estimates were based on a small number of studies with limited sample sizes (Table 1). These findings suggest a stronger association between HPV infection and ESCC, while highlighting the limited and heterogeneous evidence for EAC.

Analysis of HPV genotypes revealed that HPV-16 was the most prevalent in both ESCC and EAC (Supplementary Table S5). In PCR-based studies, HPV-16 prevalence in ESCC was 24% (95% CI: 18%−30%), with significant heterogeneity (*I*^2^ = 96.4%, *p* < 0.0001), while in EAC, it was lower at 15% (95% CI: 2%−34%; *I*^2^ = 52.0%, *p* = 0.1243). HPV-18 prevalence in ESCC was 9% (95% CI: 6%−14%), whereas the pooled prevalence for HPV-18 in EAC was not estimable with precision due to extremely wide confidence intervals and limited data (11%, 95% CI: 0%−68%). *In situ* hybridization studies were available only for ESCC, revealing a pooled HPV-16 prevalence of 34% (95% CI: 8%−65%) with marked heterogeneity (*I*^2^ = 98.9%, *p* < 0.0001). Immunohistochemistry-based analyses found an HPV-16 prevalence of 46% (95% CI: 20%−73%) in ESCC, though with significant heterogeneity (*I*^2^ = 90.0%, *p* < 0.0001). Serological studies using ELISA reported an HPV-16 prevalence of 30% (95% CI: 12%−51%) in ESCC and 23% (95% CI: 12%−37%) in EAC, though the latter was based on a single study. HPV-18 prevalence was lower in ESCC (6%, 95% CI: 0%−30%) and was not evaluable for EAC due to insufficient data.

### 3.4 Meta-regression and publication bias

A multivariable meta-regression analysis identified geographic region as a significant predictor of HPV prevalence (Table 2). Studies from East Asia had a significantly higher prevalence of HPV compared to those from Europe (coefficient = 0.2448, *p* = 0.0131). However, factors such as detection method, histological subtype (EAC vs. ESCC), and study period did not show significant associations with prevalence heterogeneity (*p* > 0.05). Individually, the moderators explained only 12.3% of the observed heterogeneity. Specifically, region accounted for approximately 9.5% of the heterogeneity, while histological subtypes explained around 1%. The other moderators, detection method and study period, had negligible effects, with slightly negative *R*^2^ values, indicating that their inclusion did not reduce heterogeneity (Table 2). These findings suggest that geographic region contributes modestly to the variability in HPV prevalence across studies, whereas study period, detection method, and histological subtype alone do not substantially explain the observed heterogeneity.

**Table 2:** Multivariable meta-regression analysis for prevalence of HPV infection in esophageal cancer.

Characteristic	Prevalence of HPV infection		
	**Meta-regression coefficient (95% CI)**	***p*** **-Value**	***R** ^2^*
Intercept	0.4368 (0.2155, 0.6581)	0.0001	
Detection method			
Polymerase chain reaction	Reference	/	−0.010
*In situ* hybridization	−0.1516 (−0.3438, 0.0406)	0.1221	
Immunohistochemistry	−0.0675 (−0.1285, 0.2636)	0.4996	
Enzyme-linked immunosorbent assay	0.0804 (−0.1591, 0.3199)	0.6582	
Other	−0.0102 (−0.2509, 0.2304)	0.9335	
Histological subtypes			
ESCC	Reference	/	0.011
EAC	−0.1941 (−0.4542, 0.0660)	0.1436	
Region			
Europe	Reference	/	0.095
Africa	0.0027 (−0.2727, 0.2782)	0.9844	
South Asia	0.0427 (−0.2298, 0.3151)	0.7589	
East Asia	0.2448 (0.0515, 0.4381)	0.0131	
Middle East and North Africa	−0.0721 (−0.3346, 0.1904)	0.5902	
South America	−0.0788 (−0.3367, 0.1792)	0.5496	
Oceania	0.1181 (−0.2347, 0.4708)	0.5118	
Southeast Asia	−0.3326 (−0.9518, 0.2866)	0.2925	
North America	0.1844 (−0.1333, 0.5022)	0.2553	
Study period			
2000–2009	Reference	/	−0.008
2010–2019	0.0455 (−0.0771, 0.1680)	0.4671	
2020–present	0.1799 (−0.0104, 0.3702)	0.3702	

**Figure 2:**
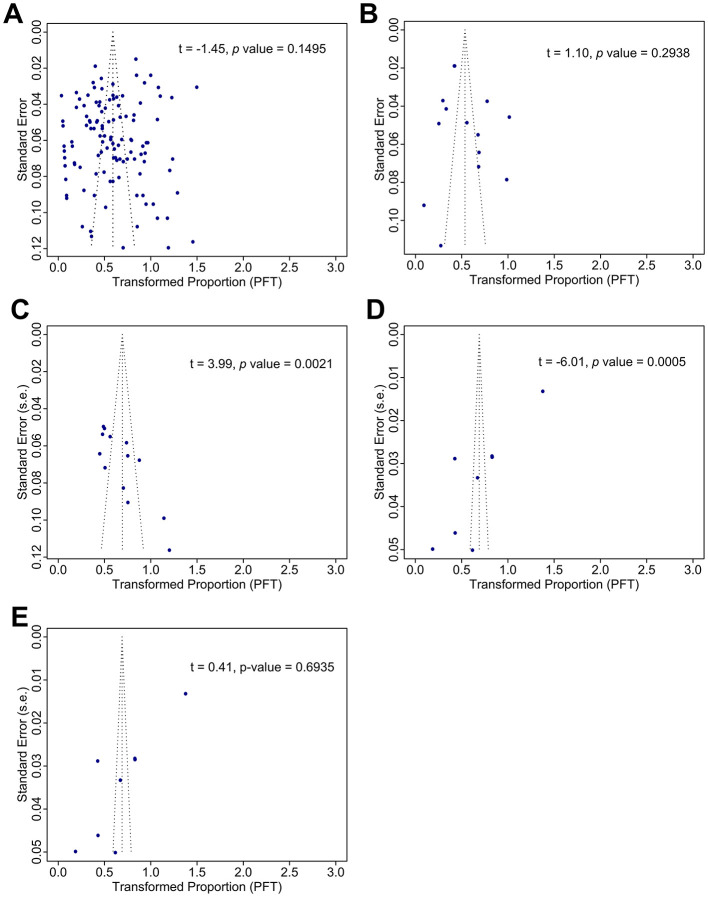
The funnel plot indicates a publication bias among the studies on the prevalence of HPV infection in esophageal cancer, categorized by detection method. The methods include Polymerase Chain Reaction **(A)**, *In Situ* Hybridization **(B)**, Immunohistochemistry **(C)**, Enzyme-Linked Immunosorbent Assay (ELISA) **(D)**, and other methods **(E)**. Potential publication bias was evaluated using both the funnel plot and Egger's test.

Publication bias was evaluated through funnel plots and Egger's test (Figure 2). For studies using PCR, the funnel plot appeared symmetrical, and Egger's test showed no evidence of small-study effects (*t* = −1.45, *p* = 0.1495), indicating a low likelihood of publication bias (Figure 2A). A similar lack of bias was found in studies using *in situ* hybridization, with no significant Egger's test results (*t* = 1.10, *p* = 0.2938, Figure 2B). Studies using other methods also showed no significant publication bias, although the small number of studies limited interpretation (Figure 2E). In contrast, studies using immunohistochemistry and ELISA showed evidence of publication bias, with asymmetric funnel plots and significant Egger's test results (immunohistochemistry: *t* = 3.99, *p* = 0.0021, Figure 2C; ELISA: *t* = −6.01, *p* = 0.0005, Figure 2D).

### 3.5 Association between HPV infection and esophageal cancer risk

**Figure 3:**
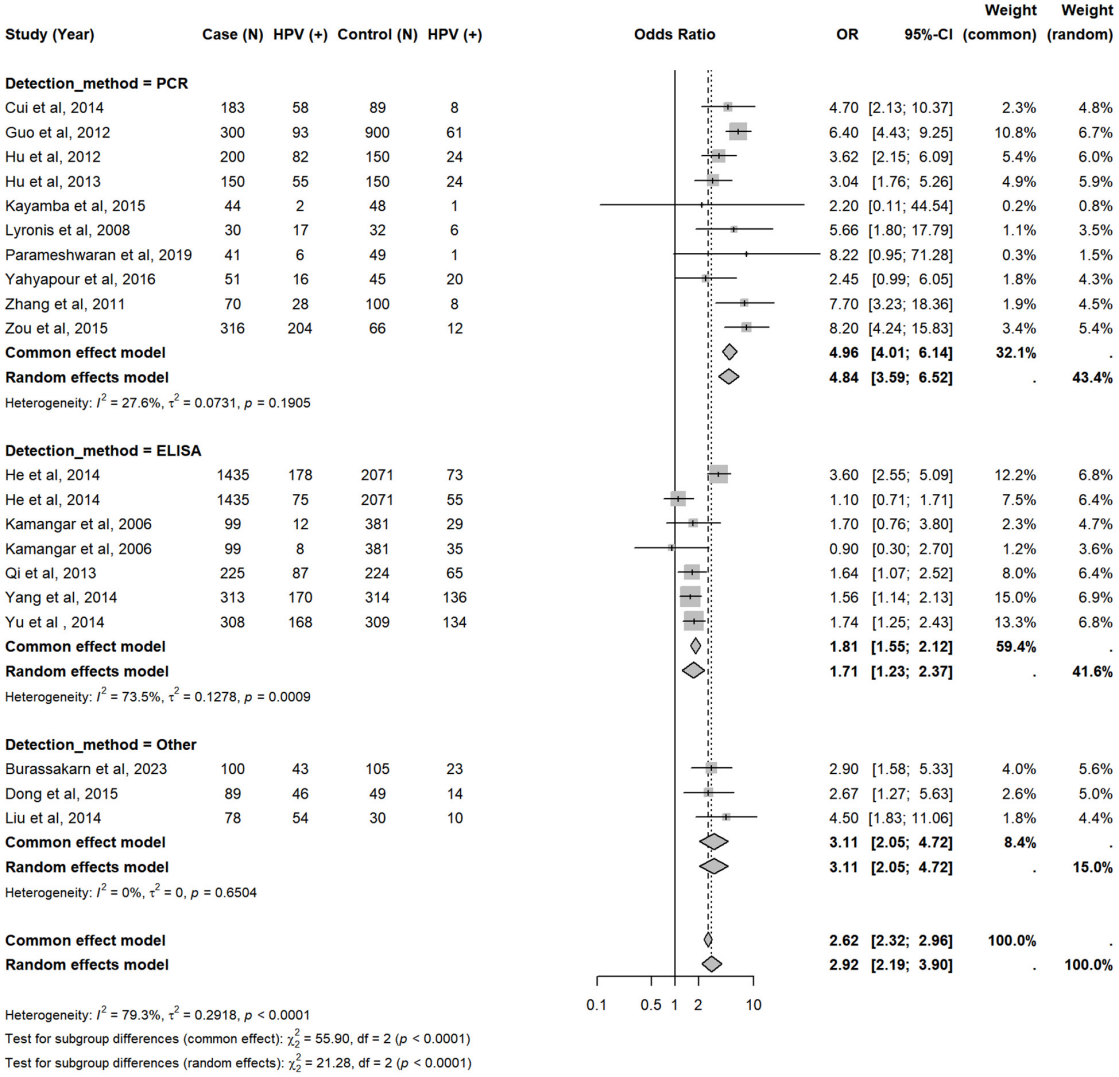
Forest plot of the overall association between HPV infection and esophageal cancer, by detection method. Each horizontal line represents a study-specific OR with 95% CI for the association between HPV infection and esophageal cancer. The pooled OR was calculated using a random-effects model. Study weights are proportional to inverse-variance and indicated on the right.

The meta-analysis of case-control studies demonstrated a significant association between HPV infection and esophageal cancer risk (Figure 3). The pooled odds ratio (OR) for all studies was 2.92 (95% CI: 2.19–3.90), indicating that HPV-positive individuals had nearly three times the risk of developing esophageal cancer compared to HPV-negative individuals (*p* < 0.0001). Subgroup analyses highlight this association for both ESCC (OR = 2.75, 95% CI: 2.05–3.70) and EAC (OR = 2.79, 95% CI: 1.20–6.48), despite the wider confidence intervals for EAC due to the smaller number of studies (Supplementary Figures S3, S4). HPV-16 infection was strongly associated with esophageal cancer risk (OR = 3.54, 95% CI: 2.38–5.25, Supplementary Figure S5), while the association with HPV-18 was not statistically significant (OR = 1.32, 95% CI: 0.91–1.91, Supplementary Figure S6).

To evaluate the robustness of our findings and address concerns regarding potential bias from lower-quality observational studies, we conducted sensitivity analyses restricted to studies assessed as high quality using the Newcastle–Ottawa Scale (NOS >7). In these analyses, the association between HPV infection and EC risk was not materially altered when only high-quality case–control studies were included. Overall, these findings indicate that the main conclusions of this meta-analysis are robust to variation in methodological quality and are not driven by studies at higher risk of bias (Supplementary Figure S7).

## 4 Discussion

This systematic review and meta-analysis provide a comprehensive update on the prevalence of HPV in EC and its association with disease risk. By synthesizing data from 151 studies involving over 18,000 cases, we report an overall pooled HPV prevalence of 31% in EC patients when detected by PCR. A critical finding of this study is the significant divergence in prevalence between histological subtypes, with HPV infection being notably more frequent in esophageal squamous cell carcinoma (ESCC, 29%) compared to esophageal adenocarcinoma (EAC, 16%). Furthermore, our meta-analysis of case-control studies demonstrating that HPV infection is associated with a nearly three-fold increase in the risk of developing EC (OR = 2.92), with HPV-16 identified as the primary high-risk genotype. Notably, most included studies were case-control or cross-sectional in design, which are inherently vulnerable to selection bias and reverse causation. Therefore, our results support an epidemiological association between HPV detection and EC risk, but cannot establish temporality or direct etiologic mechanisms.

The observed prevalence of HPV in ESCC aligns with the virus's known tropism for squamous epithelium, paralleling its established role in cervical and oropharyngeal cancers. Our estimate of 29% for ESCC is consistent with, though slightly higher than, previous meta-analyses that reported rates ranging from 18 to 25% (Petrelli et al., 2021; Mohammadpour et al., 2019). This variation likely reflects our inclusion of recent studies from high-incidence regions (Syrjänen, 2013), as our meta-regression identified East Asia as a significant predictor of higher prevalence (44%). The geographic disparity—contrasting sharply with lower rates in Europe (18%) and North America—suggests that environmental co-factors, such as dietary habits, microbiome composition, or genetic susceptibility, may modulate the viral persistence or carcinogenic potential of HPV in the esophagus (Sharma et al., 2022; Zhou et al., 2021; Baghdadi et al., 2014). The elevated HPV prevalence observed in East Asia may partly reflect the concentration of studies conducted in high-incidence ESCC areas within the Esophageal Cancer Belt, underscoring the potential interaction between geographic risk environments and HPV detection in esophageal cancer.

A novel and important contribution of this study is the estimation of HPV prevalence in EAC, a domain that has received far less attention than ESCC in prior research (Petrick et al., 2014). While previous studies have largely focused on ESCC, we observed a detectable HPV prevalence of 16% in EAC, challenging the conventional view that HPV is unrelated to adenocarcinoma pathogenesis (Antonsson et al., 2016). Although this prevalence is lower than that reported for ESCC, the pooled analysis demonstrated a statistically significant association between HPV infection and EAC risk (OR = 2.79), suggesting that HPV may contribute to carcinogenesis in a subset of cases. One possible explanation is that HPV could interact with chronic inflammatory processes associated with gastroesophageal reflux disease or Barrett's esophagus, thereby facilitating malignant transformation (Rajendra et al., 2013; Cook et al., 2014). Nevertheless, these findings should be interpreted with caution. The evidence base for esophageal adenocarcinoma (EAC) remains limited, as the number of available studies is relatively small and the pooled estimates are characterized by wide confidence intervals, reflecting substantial uncertainty. Consequently, although our results suggest a potential association between HPV and EAC, they should be regarded as hypothesis-generating rather than definitive. While HPV DNA has been detected in a subset of EAC cases and pooled analyses indicate an association with EAC risk, the current evidence is constrained by heterogeneous methodologies and a lack of consistent mechanistic validation. The limited number of EAC-specific studies, combined with these methodological inconsistencies, precludes firm conclusions regarding a biological or causal role of HPV in EAC. Future large-scale studies employing standardized HPV detection methods and validated biomarkers of biologically active infection are needed to clarify whether HPV plays an independent etiological role in EAC.

The substantial heterogeneity across studies (*I*^2^ > 90%) remains a major challenge in this field. Therefore, the pooled prevalence values presented in this meta-analysis should be interpreted as broad summary measures rather than precise universal estimates. Our meta-regression identified geographic region as an important contributor to this variability, but detection methodology also played a critical role. Specifically, immunohistochemistry (IHC) yielded higher HPV prevalence estimates (41%) than PCR (31%) and *in situ* hybridization (ISH, 26%), underscoring that methodological differences substantially influence reported prevalence. These discrepancies reflect inherent limitations of each detection approach. PCR is highly sensitive for detecting HPV DNA but cannot discriminate between transcriptionally active infection and incidental (“passenger”) viral presence, potentially leading to overestimation of oncogenic involvement, particularly in tissues where HPV is not an established causal agent (Geßner et al., 2018; Kimple et al., 2012). MY09/11 primers amplify a broader L1 region and were widely used historically, but have lower sensitivity for some genotypes, especially in formalin-fixed samples. GP5+/6+ primers target a shorter fragment, improving amplification in degraded DNA, and generally detect a wider range of high-risk HPV genotypes with higher sensitivity (Remmerbach et al., 2004). SPF10 primers amplify an even shorter region, providing the highest analytical sensitivity and allowing detection of multiple HPV types in multiplex formats (Micalessi et al., 2013). Differences in primer selection may partly explain the variation in reported HPV prevalence across studies and underscore the need to consider assay design when interpreting pooled prevalence estimates. Accordingly, we performed a stratified analysis to estimate pooled PCR-based HPV detection rates according to the reported primer systems (Supplementary Table S4). However, substantial heterogeneity persisted across nearly all primer categories (*I*^2^ > 95% for most groups), indicating that primer selection alone does not fully account for the observed variability in HPV prevalence. Several factors likely contributed to this heterogeneity: (1) inconsistent reporting of primer details across studies, particularly in earlier publications; (2) the use of multiple or combined primer systems in several studies, which hindered mutually exclusive classification; and (3) small numbers of studies within some primer categories, limiting the stability of pooled estimates.

ISH can confirm viral integration but suffers from lower sensitivity, which may underestimate true prevalence. Meanwhile, IHC—typically based on p16 overexpression—is not a validated surrogate marker of HPV-driven carcinogenesis in esophageal cancer, as p16 upregulation can arise from non-viral mechanisms such as inflammation or alternative cell-cycle dysregulation pathways (Ishida et al., 2021; Mooren et al., 2014). Consequently, IHC-based estimates are likely to overstate the role of HPV in esophageal cancer. Similarly, serological assays (e.g., ELISA) detect prior exposure to HPV rather than active infection or tumor-specific viral involvement and therefore cannot be interpreted as evidence of causality. Collectively, these findings indicate that reliance on a single detection method is insufficient to define HPV involvement in esophageal cancer. Future studies should adopt multimodal approaches—integrating HPV DNA detection (PCR), p16 IHC, and preferably E6/E7 mRNA expression or integration analyses—to more accurately identify biologically active HPV infection and clarify its etiological relevance in esophageal carcinogenesis (Zito Marino et al., 2020).

Biologically, the predominance of HPV-16 in ESCC (24%) and its strong association with cancer risk (OR = 5.27) are compatible with a potential role of the viral E6 and E7 oncoproteins in esophageal carcinogenesis through inactivation of p53 and Rb tumor suppressors (Zhang et al., 2025), a mechanism well-established in other HPV-driven malignancies (Moody and Laimins, 2010). By contrast, the absence of a significant association for HPV-18 (OR = 1.32) indicates that high-risk HPV genotypes may differ in their oncogenic activity within the esophageal microenvironment (Yong et al., 2013; Song et al., 2025). Nevertheless, epidemiological associations have not been consistently supported by molecular evidence of transcriptionally active HPV in EC. Unlike cervical and oropharyngeal cancers—where E6/E7 expression, viral integration, and consequent disruption of p53/Rb signaling are well-documented—most studies in EC have not systematically demonstrated these mechanistic hallmarks, and only a limited number have assessed E6/E7 mRNA expression or integration status. Importantly, several rigorous molecular investigations have challenged a causal role for HPV in esophageal cancer; for example, (Halec et al. 2016) found no evidence of transcriptionally active HPV or HPV-driven oncogenic signaling in esophageal tumors, arguing against HPV as a necessary driver of carcinogenesis. Collectively, these data suggest that HPV is more likely to represent a cofactor or incidental finding rather than a primary etiologic agent in many EC cases.

While most included studies relied on HPV DNA detection, which may capture incidental viral presence, a subset evaluated more biologically informative markers, including E6/E7 mRNA expression and viral integration, which are more suggestive of transcriptionally active and potentially oncogenic infection. In our supplementary analysis of 10 eligible studies (*n* = 868), the pooled prevalence of HPV positivity was 30% among studies assessing E6/E7 mRNA and 61% among those evaluating viral integration, corresponding to an overall prevalence of 42%. These findings suggest that biologically active or integrated HPV may be present in a proportion of esophageal cancer cases; however, the limited number of studies and substantial heterogeneity preclude definitive conclusions regarding causality. While the detection of E6/E7 mRNA expression or viral integration may suggest possible biological activity of HPV, these findings remain indirect and do not establish HPV as a causal or driving factor in esophageal carcinogenesis. Moreover, pooled estimates based on E6/E7 mRNA expression and viral integration should be regarded as exploratory and primarily contextual rather than definitive quantitative inference, given the imprecision and variability across studies. Although these markers are more indicative of transcriptionally active HPV infection, the current evidence base remains insufficient to establish a causal role for HPV in esophageal carcinogenesis. Collectively, these results should be interpreted as hypothesis-generating and underscore the need for larger, well-designed studies employing standardized multimodal HPV detection strategies that integrate HPV DNA testing with E6/E7 transcript assays, integration analyses, and relevant host biomarkers. Accordingly, these findings should be regarded as hypothesis-generating rather than confirmatory, and they do not establish HPV as a causal driver of esophageal cancer.

In contrast, the role of HPV in EAC is less straightforward because EAC arises from glandular epithelium, a tissue type not considered a primary target of HPV infection. This raises important questions regarding how HPV could contribute to malignant transformation in this context. One potential explanation is that HPV infection may occur in areas of metaplastic or dysplastic epithelium, such as Barrett's esophagus, where squamous–columnar junctional or transitional cell populations might be more permissive to viral entry and persistence (Rajendra et al., 2013; Rajendra and Sharma, 2023). Alternatively, HPV could act indirectly by promoting a pro-inflammatory microenvironment, genomic instability, or epigenetic alterations that facilitate neoplastic progression in predisposed tissues rather than serving as a direct oncogenic driver (Rajendra and Sharma, 2023). Another possibility is that HPV detection in EAC reflects incidental or passenger infection of adjacent squamous or metaplastic cells rather than true infection of glandular tumor cells (Iyer et al., 2011). Although HPV DNA has been detected in a subset of EAC specimens, the evidence remains limited and lacks robust mechanistic validation. Accordingly, any putative role of HPV in esophageal adenocarcinoma pathogenesis should be interpreted with caution and requires confirmation in well-designed prospective and molecular studies.

Several limitations must be acknowledged. First, the substantial heterogeneity observed suggests that unmeasured confounders, such as sexual behavior, smoking, and alcohol consumption (Del Pino et al., 2024), could not be fully adjusted for in the meta-regression due to inconsistent reporting across primary studies. Although we explored heterogeneity using subgroup analyses and meta-regression, many potentially important contributors (such as specimen type, FFPE processing, viral gene targets, primer sets, and contamination prevention measures) could not be systematically evaluated because these details were inconsistently reported across primary studies. As a result, residual heterogeneity remained high even after adjustment for region and detection method. However, our sensitivity analyses restricted to high-quality studies (NOS > 7) produced results comparable to the main analysis. This suggests that the observed association between HPV infection and EC risk is not solely explained by study quality differences, although causality cannot be definitively established. Moreover, limited availability of studies assessing biologically active HPV infection, including E6/E7 mRNA expression and viral integration. The sparse data and high heterogeneity observed in these analyses restrict the interpretability of pooled estimates. Future investigations should adopt standardized reporting frameworks and incorporate multimodal confirmation of biologically active HPV infection (e.g., DNA PCR combined with E6/E7 mRNA expression or ISH) to improve comparability across studies. Second, the potential for publication bias was evident in studies utilizing IHC and ELISA, possibly leading to an overestimation of prevalence in those subgroups. Third, although our analysis provides additional insights into EAC, the number of studies examining this subtype remains limited relative to ESCC, which reduces the precision of our estimates. Moreover, evidence supporting HPV involvement in EAC is sparse, and only a small number of studies have assessed biologically active infection using markers such as E6/E7 mRNA expression or viral integration. Consequently, current findings should be regarded as exploratory and are insufficient to establish HPV as an etiologic factor in EAC. Forth, although we searched three major international databases (PubMed, Scopus, and Web of Science), additional databases such as Embase and regional platforms (e.g., African journals online) were not included. Therefore, some relevant studies, particularly from low- and middle-income settings, may have been missed.

Although prophylactic HPV vaccination has substantially reduced the burden of cervical and other HPV-related cancers, its potential impact on esophageal cancer remains uncertain and should be interpreted with caution. Our findings suggest that HPV accounts for only a modest proportion of esophageal cancer cases—primarily in ESCC—and current evidence is insufficient to justify vaccination specifically for esophageal cancer prevention. Any potential benefit would depend on regional HPV prevalence, the fraction of tumors truly driven by oncogenic HPV rather than passenger infection, and the underlying distribution of ESCC vs. EAC. From a public-health perspective, HPV vaccination may confer ancillary benefits for esophageal cancer in high-incidence settings if a causal role is confirmed, but its cost-effectiveness would almost certainly be determined by established outcomes, such as prevention of cervical and oropharyngeal cancers. To inform policy, future research should prioritize large prospective cohorts to establish temporality, mechanistic studies assessing E6/E7 expression, p16 concordance, and viral integration in both ESCC and EAC, and region-specific modeling to estimate potential vaccine impact.

## 5 Conclusions

Epidemiological evidence indicates that HPV infection—particularly HPV-16—is associated with esophageal cancer, with higher detection rates observed in ESCC than in EAC. However, existing data—largely derived from observational studies and lacking robust mechanistic validation—are insufficient to establish HPV as a definitive causal driver of esophageal carcinogenesis, particularly for EAC. To determine the biological relevance of HPV in EC, future research should systematically evaluate E6/E7 expression and viral integration status. In addition, well-designed longitudinal studies are needed to clarify the temporal relationship between HPV infection and esophageal cancer development and to assess whether HPV status has potential value as a prognostic biomarker in EC patients.

## Data Availability

The original contributions presented in the study are included in the article/Supplementary material, further inquiries can be directed to the corresponding author.
