# Supplementary material for: HPV prevalence in esophageal cancer: an updated systematic review and meta-analysis
Source: Front Microbiol. 2026 Apr 7;17:1771173. doi: 10.3389/fmicb.2026.1771173 (PMC13095747; doi:10.3389/fmicb.2026.1771173)
Supplement: Supplementary file 1 [file Data_Sheet_1.pdf]

**Supplementary Table S1.** Characteristics of included studies evaluating HPV prevalence in esophageal cancer patients and the association between HPV infection and esophageal cancer, stratified by HPV detection method

| Study (Year Published) | Region | Country | HPV DNA Source | Detection method | HPV genotype | PCR Primers or Serology Antibodies | No. of EC Cases | No. of EC Cases with HPV (+) | No. of Control | No. of Control with HPV (+) | OR | LCI | UCI |
|---|---|---|---|---|---|---|---|---|---|---|---|---|---|
| *Reynders et al*, 2025 | Europe | Belgium | FFPE | Multiplex PCR | Any | Not report | 226 ESCC | 28 | / | / | / | / | / |
| | | | | | | | 152 EAC | 48 | / | / | / | / | / |
| *de Oliveira et al*, 2025 | Europe | Northern Ireland | FFPE | Multiplex PCR | Any | E7 | 150 EAC | 11 | / | / | / | / | / |
| *Qu et al, 2024* | East Asia | China | FFPE | In Situ Hybridization | HPV 16 | / | 178 ESCC | 87 | / | / | / | / | / |
| *Ndemela et al, 2024* | Africa | Tanzania | FFPE | Conventional PCR | Any | MY09/11, HPV 16 Specific, HPV 18 Specific | 107 ESCC | 55 | / | / | / | / | / |
| | | | | | | | 11 EAC | 8 | / | / | / | / | / |
| *Kotian et al, 2024* | South Asia | India | FFT | Conventional PCR | HPV 16/18 | HPV 16 Specific, HPV 18 Specific | 30 ESCC | 4 | / | / | / | / | / |
| Yuan et al, 2023 | East Asia | China | FFPE | Conventional PCR | HPV 16/18 | GP5+/6+, HPV 16 Specific, HPV 18 Specific | 1112 ESCC | 616 | / | / | / | / | / |
| *Maroga et al* | Africa | South Africa | FFPE | Conventional PCR | Any | L1 | 50 ESCC | 45 | / | / | / | / | / |
| *Burassakarn et al, 2023* | Southeast Asia | Thailand | FFPE | Direct Flow CHIP system | Any | Not report | 72 ESCC | 32 | 105 | 23 | 3.2 | 1.2 | 6.1 |
| | | | | | | | 28 EAC | 11 | | | 2.3 | 0.9 | 5.6 |
| Sadeghian et al, 2022 | Middle East and North Africa | Iran | FFPE | Conventional PCR | Any | MY09/11, GP5+/6+ | 35 ESCC | 14 | 70 | 0 | / | / | / |
| | | | | | | | 35 EAC | 6 | | | / | / | / |
| Munari et al, 2022 | South America | Brazil | FFPE | Multiplex PCR | Any | Not report | 51 ESCC | 17 | / | / | / | / | / |
| Zhang et al, 2021 | Asia | China | Serum | Conventional PCR | Any | MY09/11, GP5+/6+ | 105 | 33 | 60 | 6 | / | / | / |

| | | | | | | | | | | | | | |
|---|---|---|---|---|---|---|---|---|---|---|---|---|---|
| Woellner et al, 2021 | South America | Brazil | FFPE | Immunohistochemistry | High-risk HPV | / | 58 ESCC | 27 | / | / | / | / | / |
| Ishida et al, 2020 | East Asia | Japan | FFPE | Immunohistochemistry | High-risk HPV | / | 82 ESCC | 7 | / | / | / | / | / |
| | | | | | | | 15 Small-cell carcinomas | 15 | / | / | / | / | / |
| Zheng et al, 2020 | East Asia | China | FFPE | Conventional PCR | Any | GP5+/6+ | 54 ESCC | 33 | / | / | / | / | / |
| Zheng et al, 2020 | East Asia | China | FFPE | Immunohistochemistry | HPV 16 | / | 73 ESCC | 33 | / | / | / | / | / |
| Sultana et al, 2020 | South Asia | Pakistan | FFPE | Conventional PCR | Any | GP5+/6+, HPV 16 Specific, HPV 18 Specific | 203 ESCC | 63 | / | / | / | / | / |
| Singh et al, 2020 | South Asia | India | FFPE | Immunohistochemistry | HPV 16 | / | 30 ESCC | 14 | / | / | / | / | / |
| Kanaan et al, 2020 | Europe | France | FFPE | Immunohistochemistry | HPV 16 | / | 86 ESCC | 18 | / | / | / | / | / |
| Inoue et al, 2020 | East Asia | Japan | FFPE | In Situ Hybridization | HPV 16/18 | / | 145 ESCC | 15 | / | / | / | / | / |
| Dinc et al, 2020 | Middle East and North Africa | Turkey | FFPE | Conventional PCR | HPV16 or others | Not report | 21 ESCC | 12 | / | / | / | / | / |
| Parameshwaran et al, 2019 | Oceania | Australia | FFT | Conventional PCR | HPV 16/18 | HPV 16 Specific, HPV 18 Specific | 41 EAC | 9 | 49 | 9 | / | / | / |
| | | | Serum | Droplet digital PCR | HPV 16/18 | HPV 16 Specific, HPV 18 Specific | 41 EAC | 6 | 49 | 1 | 8.22 | 0.95 | 71.45 |
| Leon et al, 2019 | Africa | Ethiopia | FFT | Multiplex PCR | Any | Not report | 62 | 1 | 56 | 4 | / | / | / |
| Baş et al, 2019 | Middle East and North Africa | Turkey | FFPE | Conventional PCR | High-risk HPV | Not report | 47 ESCC | 1 | / | / | / | / | / |
| | Africa | Somalia | | | | | 45 ESCC | 0 | / | / | / | / | / |
| Vošmik et al, 2018 | Europe | Czech Republic | FFPE | Next-generation Sequencing | Any | Not report | 37 EAC | 0 | / | / | / | / | / |
| Li et al, 2018 | East Asia | China | FFT | Conventional PCR | Any | MY09/11, HPV 16 Specific | 189 ESCC | 169 | / | / | / | / | / |
| Ghaffar et al, 2018 | East Asia | China | Not report | Conventional PCR | Any | MY09/11, GP5+/6+, HPV 16 Specific, HPV 18 Specific | 66 | 45 | / | / | / | / | / |
| Geßner et al, 2018 | Africa | Malawi | FFPE | Conventional PCR | HPV16/18/31/45 | LCR/E6/E7 | 40 ESCC | 6 | 12 | 0 | / | / | / |
| da Costa et al, 2018 | South America | Brazil | FFT | Conventional PCR | High Risk HPV | Not report | 87 ESCC | 12 | 87 | 12 | / | / | / |
| Cheah et al, 2018 | Southeast | Malaysia | FFPE | Conventional PCR | Any | Not report | 67 ESCC | 1 | / | / | / | / | / |

| | | | | | | | | | | | | | |
|---|---|---|---|---|---|---|---|---|---|---|---|---|---|
| | Asia | | | | | | | | | | | | |
| Zhang et al, 2017 | East Asia | China | FFPE | Conventional PCR | HPV 16/18 | HPV 16 Specific, HPV 18 Specific | 192 ESCC | 67 | / | / | / | / | / |
| Rajendra et al, 2017 | Oceania | Australia | FFT | Conventional PCR | Any | MY09/11, GP5+/6+ | 65 EAC | 20 | / | / | / | / | / |
| Pastrez et al, 2017 | South America | Brazil | FFT | Conventional PCR | Any | Not report | 87 ESCC | 10 | 87 | 9 | / | / | / |
| Chan et al, 2017 | East Asia | China | FFPE | Conventional PCR | Any | MY09/11, GP5+/6+ | 143 ESCC | 5 | 168 | 9 | / | / | / |
| Yahyapour et al, 2016 | Middle East and North Africa | Iran | FFPE | Conventional PCR | Any | MY09/11 | 51 ESCC | 16 | 45 | 20 | 2.45 | 1.01 | 6.16 |
| Soheili et al, 2016 | Middle East and North Africa | Iran | FFPE | Conventional PCR | Any | GP5+/6+ | 103 ESCC | 11 | / | / | / | / | / |
| Prakash et al, 2016 | South Asia | India | FFPE | Hybrid DNA capture technique | High-risk HPV | / | 18 ESCC | 9 | / | / | / | / | / |
| Antonsson et al, 2016 | Oceania | Australia | FFPE | Conventional PCR | Any | GP5+/6+ | 201 EAC | 0 | / | / | / | / | / |
| Zou et al, 2015 | East Asia | China | FFPE | Conventional PCR | Any | GP5+/6+, E7 | 316 ESCC | 204 | 66 | 12 | 8.196 | 4.28 | 15.964 |
| Wang et al, 2015 | East Asia | China | FFPE | Conventional PCR | Any | MY11, GP6+ | 150 ESCC | 27 | / | / | / | / | / |
| Mehryar et al, 2015 | East Asia | China | FFPE | Conventional PCR | Any | GP5+/6+, HPV 16 Specific, HPV 18 Specific | 198 ESCC | 158 | / | / | / | / | / |
| Kumar et al, 2015 | South Asia | India | FFPE | Immunohistochemistry | High-risk HPV | / | 101 ESCC | 22 | / | / | / | / | / |
| Kayamba et al, 2015 | Africa | Zambia | FFT | Conventional PCR | Any | L1 | 44 ESCC | 2 | 48 | 1 | 2.2 | 0.1 | 41 |
| Georgantis et al, 2015 | Europe | Greece | FFT | Conventional PCR | Any | MY09/11 | 19 ESCC | 2 | 30 | 0 | / | / | / |
| Dong et al, 2015 | East Asia | China | FFPE | MassARRAY MALDI-TOF MS | Any | E6, L1 | 89 ESCC | 46 | 49 | 14 | 2.67 | 1.27 | 5.64 |
| Zhang et al, 2014 | East Asia | China | FFT | Not report | Any | L1, HPV 16/18/58 | 70 ESCC | 43 | 50 | 14 | / | / | / |
| Yu et al, 2014 | East Asia | China | Serum | ELISA | HPV16 | L1 | 308 ESCC | 168 | 309 | 134 | 1.74 | 1.25 | 2.43 |
| Yang et al, 2014 | East Asia | China | Serum | ELISA | HPV16 | L1 | 313 ESCC | 170 | 314 | 136 | 1.56 | 1.14 | 2.13 |
| Teng et al, 2014 | East Asia | China | FFPE | PCR and reverse dot blot | Any | L1 | 177 ESCC | 6 | / | / | / | / | / |
| Ludmir et al, 2014 | North America | USA | FFPE | In Situ Hybridization | HPV16/18 | / | 19 ESCC | 1 | / | / | / | / | / |
| Liu et al, 2014 | East Asia | China | FFPE | PCR and hybridization | Any | / | 78 ESCC | 60 | 30 | 11 | 4.5 | 1.83 | 11.05 |

| | | | | | | | | | | | | | |
|---|---|---|---|---|---|---|---|---|---|---|---|---|---|
| He et al, 2014 | East Asia | China | Serum | ELISA | HPV 16/18/57 | E7 | 1435 ESCC | 1382 | 2071 | 2063 | / | / | / |
| Cui et al, 2014 | East Asia | China | FFPE | Conventional PCR | Any | MY09/11 | 183 ESCC | 58 | 89 | 8 | 4.7 | 2.13 | 10.36 |
| Chen et al, 2014 | East Asia | China | FFT | Conventional PCR | Any | MY09/11, GP5+/6+ | 66 ESCC | 44 | 66 | 8 | / | / | / |
| Cao et al, 2014 | East Asia | China | FFPE | In Situ Hybridization | HPV16/18 | / | 105 ESCC | 29 | / | / | / | / | / |
| Yang et al, 2013 | East Asia | China | Serum | ELISA | HPV 16 | L1 | 307 ESCC | 167 | 311 | 135 | / | / | / |
| Yahyapour et al, 2013 | Middle East and North Africa | Iran | FFPE | Conventional PCR | Any | MY09/11 | 177 ESCC | 49 | / | / | / | / | / |
| Wang et al, 2013 | East Asia | China | FFT | Conventional PCR | Any | MY09/11 | 92 ESCC | 19 | 40 | 0 | / | / | / |
| Vaiphei et al, 2013 | South Asia | India | FFT | Conventional PCR | Any | L1 | 23 ESCC | 20 | 23 | 0 | / | / | / |
| Schäfer et al, 2013 | Africa | South Africa | FFT | Conventional PCR | Any | MY09/11, GP5+/6+ | 114 ESCC | 10 | / | / | / | / | / |
| Rajendra et al, 2013 | Oceania | Australia | FFT | Conventional PCR | Any | MY09/11, GP5+/6+, HPV 16 Specific, HPV 18 Specific | 27 EAC | 18 | 122 | 22 | / | / | / |
| Qi et al, 2013 | East Asia | China | Serum | ELISA | / | / | 225 ESCC | 87 | 224 | 65 | 1.64 | 1.07 | 2.52 |
| Mohiuddin et al, 2013 | South Asia | India | FFT | Conventional PCR | Any | Not report | 56 ESCC | 11 | 59 | 27 | / | / | / |
| Liu et al, 2013 | East Asia | China | FFPE | Conventional PCR | Any | GP5+/6+ | 253 ESCC | 52 | / | / | / | / | / |
| Hu et al, 2013 | East Asia | China | FFPE | Conventional PCR | HPV 16 | HPV 16 Specific | 150 ESCC | 55 | 150 | 24 | 3.039 | 1.756 | 5.26 |
| Haeri et al, 2013 | Middle East and North Africa | Iran | FFPE | Conventional PCR | Any | GP5+/6+ | 30 ESCC | 0 | 30 | 0 | / | / | / |
| Feng et al, 2013 | East Asia | China | FFPE | Conventional PCR | Any | GP5+/6+, CPI/CPIIG, SPF10, pU-1M/pU2R, and pU31B/pU2R | 57 ESCC | 0 | / | / | / | / | / |
| Antunes et al, 2013 | South America | Brazil | FFPE | Conventional PCR | Any | MY09/11, GP5+/6+ | 51 ESCC | 0 | 37 | 0 | / | / | / |
| Qu et al, 2012 | East Asia | China | FFT | Conventional PCR | Any | GP5+/6+, HPV 16 Specific, HPV 18 Specific | 23 ESCC | 19 | / | / | / | / | / |
| Noori et al, 2012 | Middle East and North Africa | Iran | FFPE | Conventional PCR | Any | L1, HPV 16 Specific, HPV 18 Specific | 92 ESCC | 0 | 20 | 0 | / | / | / |
| Löfdahl et al, 2012 | Europe | Sweden | FFPE | Conventional PCR | Any | GP5+/6+ | 204 ESCC | 20 | / | / | / | / | / |

| | | | | | | | | | | | | | |
|---|---|---|---|---|---|---|---|---|---|---|---|---|---|
| Hu et al, 2012 | East Asia | China | FFPE | Conventional PCR | HPV 16 | HPV 16 Specific | 200 ESCC | 82 | 150 | 24 | 3.62 | 2.15 | 6.09 |
| Herbster et al, 2012 | South America | Brazil | FFPE | Conventional PCR | Any | MY09/11, GP5+/6+ | 264 ESCC | 38 | / | / | / | / | / |
| Gupta et al, 2012 | South Asia | India | Air-dried MGG-stained crush smears | Conventional PCR | Any | MY09/11 | 44 ESCC | 17 | / | / | / | / | / |
| | | | | | | | 5 EAC | 0 | / | / | / | / | / |
| Guo et al, 2012 | East Asia | China | FFT | Conventional PCR | Any | SPF1/GP6+ | 300 ESCC | 93 | 900 | 61 | 6.4 | 4.4 | 9.2 |
| Dąbrowski et al, 2012 | Europe | Poland | FFT | Conventional PCR | Any | MY09/11, HPV 16 Specific, HPV 18 Specific | 56 ESCC | 28 | 35 | 4 | / | / | / |
| Abdirad et al, 2012 | Middle East and North Africa | Iran | FFPE | Conventional PCR | Any | SPF10 | 93 ESCC | 8 | / | / | / | / | / |
| Zhang et al, 2011 | East Asia | China | FFPE | Conventional PCR | Any | L1, HPV 16 Specific, HPV 18 Specific | 106 | 82 | 100 | 33 | | | |
| Zhang et al, 2011 | East Asia | China | FFPE | Conventional PCR | HPV 16 | HPV 16 specific | 70 | 28 | 100 | 8 | 7.7 | 3.2 | 18.2 |
| Malik et al, 2011 | North America | USA | FFPE | Immunohistochemistry | High-risk HPV | / | 25 ESCC | 21 | / | / | / | / | / |
| Iyer et al, 2011 | North America | USA | FFPE | Conventional PCR | Any | GP5+/6+ | 36 EAC | 11 | 29 | 7 | / | / | / |
| | | | | Immunohistochemistry | High-risk HPV | / | 36 EAC | 15 | 29 | 6 | / | / | / |
| Hussain et al, 2011 | South Asia | India | FFT | Conventional PCR | Any | MY09/11, HPV 16 Specific, HPV 18 Specific | 75 ESCC | 14 | / | / | / | / | / |
| Goto et al, 2011 | East Asia | Japan, Korea, China | FFPE | Conventional PCR | Any | GP5+/6+ | 181 | 9 | / | / | / | / | / |
| | | | | In Situ Hybridization | Any | / | 181 | 15 | / | / | / | / | / |
| Cui et al, 2011 | East Asia | China | FFPE | Conventional PCR | Any | MY09/11, GP5+/6+, HPV 16 Specific, HPV 18 Specific | 18 ESCC | 18 | / | / | / | / | / |
| Castillo et al, 2011 | East Asia | Japan | FFPE | Conventional PCR | Any | SPF1/2 | 75 ESCC | 11 | / | / | / | / | / |
| | South Asia | Pakistan | | | | | 42 ESCC | 11 | / | / | / | / | / |
| | South America | Colombia | | | | | 49 ESCC | 9 | / | / | / | / | / |
| Ayshamgul et al, 2011 | East Asia | China | FFPE | Conventional PCR | HPV 16 | HPV 16 Specific | 50 | 19 | / | / | / | / | / |

| | | | | | | | | | | | | | |
|---|---|---|---|---|---|---|---|---|---|---|---|---|---|
| Zhang et al, 2010 | East Asia | China | FFT | Conventional PCR | HPV 16/18/58 | E6/7, E2 | 70 ESCC | 35 | 60 | 20 | | | |
| | | | | Immunohistochemistry | HPV 16 | / | 54 ESCC | 32 | | | | | |
| Wang et al, 2010 | East Asia | China | FFT | Conventional PCR | Any | SPF1/GP6+ | 347 ESCC | 190 | / | / | / | / | / |
| | North America | USA | | | | | 88 | 54 | / | / | / | / | / |
| Liu et al, 2010 | East Asia | China | FFT | Conventional PCR | HPV 16 | E6 | 69 ESCC | 35 | 32 | 2 | / | / | / |
| Koshiol et al, 2010 | East Asia | China | FFT | Conventional PCR | Any | PGMY, HPV 16 Specific, HPV 18 Specific | 267 ESCC | 266 | / | / | / | / | / |
| Antonsson et al, 2010 | Oceania | Australia | FFPE | Conventional PCR | Any | GP5+/6+ | 222 ESCC | 8 | 55 | 0 | / | / | / |
| Zhao et al, 2009 | East Asia | China | FFPE | Conventional PCR | Any | GP5+/6+ | 42 | 37 | / | / | / | / | / |
| Tornesello et al, 2009 | Europe | Italy | FFPE | Conventional PCR | Any | MY09/11, GP5+/6+, CP65/70, CP66/69, FAP59/64, HPV 16 Specific, HPV 38 Specific | 36 ESCC | 10 | / | / | / | / | / |
| | | | | | | | 20 EAC | 2 | / | / | / | / | / |
| Liao et al, 2009 | East Asia | China | FFPE | Conventional PCR | HPV 16 | E6 | 63 | 23 | 126 | 21 | / | / | / |
| Herrera-Goepfert et al, 2009 | North America | Mexico | FFPE | Conventional PCR | Amy | L1C1/L1C2, MY09/11, GP5+/6+, HPV 16 Specific, HPV 18 Specific | 60 ESCC | 15 | / | / | / | / | / |
| Bellizzi et al, 2009 | North America | USA | FFPE | In Situ Hybridization | High-risk HPV | In Situ Hybridization | 29 ESCC | 0 | / | / | / | / | / |
| Yang et al, 2008 | East Asia | China | FFPE | Conventional PCR | HPV 16/18 | E7 | 435 ESCC | 308 | 550 | 253 | / | / | / |
| Lyronis et al, 2008 | Europe | Greece | FFPE | Conventional PCR | Amy | GP5+/6+ | 30 ESCC | 17 | 32 | 6 | 5.66 | 1.8 | 17.79 |
| Lu et al, 2008 | East Asia | China | FFPE | Conventional PCR | Amy | SPF10 | 67 ESCC | 14 | / | / | / | / | / |
| Li et al, 2008 | East Asia | China | FFT | Conventional PCR | Amy | GP5+/6+, HPV 16 Specific, HPV 18 Specific | 31 | 29 | / | / | / | / | / |
| Koh et al, 2008 | East Asia | Korea | FFPE | Conventional PCR | Amy | E6, E7, HPV 16 Specific | 102 ESCC | 0 | / | / | / | / | / |
| Gábor et al, 2008 | Europe | Hungary | FFPE | Conventional PCR | HPV 16/18 | GP5+/6+, HPV 16 Specific, HPV 18 Specific | 26 | 6 | / | / | / | / | / |
| Zhou et al, 2007 | East Asia | China | FFPE | Conventional PCR | HPV 16 | E7 | 161 ESCC | 97 | / | / | / | / | / |

| | | | | | | | | | | | | | |
|---|---|---|---|---|---|---|---|---|---|---|---|---|---|
| Shuyama et al, 2007 | East Asia | China | FFPE | Conventional PCR | Amy | GP5+/6+, SPF10 | 59 ESCC | 19 | / | / | / | / | / |
| Pantelis et al, 2007 | Europe | Germany | FFPE | Conventional PCR | HPV 16/18 | HPV 16 Specific, HPV 18 Specific | 53 ESCC | 9 | / | / | / | / | / |
| Mir et al, 2007 | South Asia | India | FFT | Conventional PCR | Amy | MY09/11, GP5+/6+ | 62 ESCC | 0 | / | / | / | / | / |
| Matsha et al, 2007 | Africa | South Africa | FFPE | Conventional PCR | Amy | MY09/11, GP5+/6+ | 114 ESCC | 51 | / | / | / | / | / |
| Liu et al, 2007 | East Asia | China | FFT | Conventional PCR | HPV 16/18 | HPV 16 Specific, HPV 18 Specific | 112 ESCC | 43 | / | / | / | / | / |
| Far et al, 2007 | Middle East and North Africa | Iran | FFPE | Conventional PCR | Amy | GP5+/6+ | 140 ESCC | 33 | / | / | / | / | / |
| Dai et al, 2007 | East Asia | China | FFT | Conventional PCR | Amy | Not report | 100 | 11 | / | / | / | / | / |
| | | | Serum | ELISA | HPV 16 | E6, E7 | 100 | 3 | / | / | / | / | / |
| Yao et al, 2006 | East Asia | China | FFPE | In Situ Hybridization | HPV 16/18 | / | 82 ESCC | 32 | / | / | / | / | / |
| | | | | Immunohistochemistry | HPV 16/18 | / | 82 ESCC | 23 | / | / | / | / | / |
| Souto Damin et al, 2006 | South America | Brazil | FFPE | Conventional PCR | Amy | GP5+/6+, HPV 16 Specific, HPV 18 Specific | 165 ESCC | 26 | / | / | / | / | / |
| Qi et al, 2006 | East Asia | China | FFPE | Immunohistochemistry | HPV 16/18 | / | 60 ESCC | 11 | / | / | / | / | / |
| | | | | In Situ Hybridization | HPV 16/18 | / | 60 ESCC | 24 | / | / | / | / | / |
| Kamangar et al, 2006 | East Asia | China | Serum | ELISA | HPV 16/18/73 | L1, L2 | 99 ESCC | 33 | / | / | / | / | / |
| Castillo et al, 2006 | South America | Colombia and Chile | FFPE | Conventional PCR | Any | GP5+/6+ | 73 ESCC | 21 | / | / | / | / | / |
| Zhu et al, 2005 | East Asia | China | FFPE | In Situ Hybridization | HPV 16 | / | 119 ESCC | 86 | / | / | / | / | / |
| White et al, 2005 | Africa | Kenya | FFPE | Conventional PCR | Any | L1 | 29 ESCC | 0 | / | / | / | / | / |
| Lyronis et al, 2005 | Europe | Greece | FFPE | Conventional PCR | Any | GP5+/6+ | 30 ESCC | 17 | 27 | 6 | / | / | / |
| Liu et al, 2005 | East Asia | China | FFPE | Conventional PCR | HPV 16 | E6 | 40 ESCC | 24 | 15 | 4 | / | / | / |
| Katiyar et al, 2005 | South Asia | India | FFT | Conventional PCR | Any | MY09/11, HPV 16 Specific, HPV 18 Specific | 101 | 27 | 26 | 2 | / | / | / |
| Farhadi et al, 2005 | Middle East and North Africa | Iran | FFPE | Conventional PCR | Amy | MY09/11, HPV 16 Specific, HPV 18 Specific | 38 ESCC | 14 | 38 | 5 | / | / | / |
| Cao et al, 2005 | East Asia | China | FFPE | Conventional PCR | Amy | GP5+/6+, HPV 16 | 265 ESCC | 207 | 357 | 203 | / | / | / |

| | | | | | | | | | | | | | |
|---|---|---|---|---|---|---|---|---|---|---|---|---|---|
| | | | | | | Specific, HPV 18 Specific | | | | | | | |
| Bahnassy et al, 2005 | Middle East and North Africa | Egypt | FFPE | Conventional PCR | Amy | SPF10 | 50 | 27 | 50 | 12 | / | / | / |
| Xu et al, 2004 | East Asia | China | FFPE | Immunohistochemistry | HPV 16 | E6, E7 | 18 ESCC | 16 | 140 | 87 | / | / | / |
| Si et al, 2004 | East Asia | China | FFT | Conventional PCR | Amy | MY09/11, HPV 16 Specific, HPV 18 Specific | 87 ESCC | 18 | / | / | / | / | / |
| Lu et al, 2004 | East Asia | China | FFPE | Conventional PCR | HPV 16 | E6 | 104 ESCC | 55 | 104 | 41 | / | / | / |
| Acevedo-Nuño et al, 2004 | North America | Mexico | FFPE | Conventional PCR | Amy | CpI/IIG | 17 ESCC | 15 | / | / | / | / | / |
| Zhou et al, 2003 | East Asia | China | FFPE | Conventional PCR | HPV 16 | E6 | 48 ESCC | 31 | 23 | 8 | / | / | / |
| | | | | In Situ Hybridization | HPV 16 | / | 48 ESCC | 19 | 23 | 6 | / | / | / |
| | | | | Immunohistochemistry | HPV 16 | / | 48 ESCC | 11 | 23 | 1 | / | / | / |
| Xu et al, 2003 | East Asia | China | FFPE | In Situ Hybridization | HPV 16 | / | 40 ESCC | 28 | / | / | / | / | / |
| Weston et al, 2003 | South America | Brazil | Not report | Hybrid Capture II | Any | / | 40 ESCC | 1 | 10 | 1 | / | / | / |
| Van Doornum et al, 2003 | Europe | Netherlands | Serum | ELISA | HPV 16 | L1 | 56 ESCC | 8 | / | / | / | / | / |
| | | | | | | | 48 EAC | 11 | / | / | / | / | / |
| Si et al, 2003 | East Asia | China | FFT and FFPE | Conventional PCR | Amy | MY09/11, HPV 16 Specific, HPV 18 Specific | 319 ESCC | 43 | / | / | / | / | / |
| Liu et al, 2003 | 2003 | Asia | FFPE | Conventional PCR | HPV 6/11/16/18/31/33 | L1 | 128 ESCC | 26 | / | / | / | / | / |
| Awerkiew et al, 2002 | Europe | Germany | FFT | Conventional PCR | Amy | GP5+/6+, A5/10, A6/8 | 23 ESCC | 0 | / | / | / | / | / |
| | | | | | | | 14 EAC | 0 | / | / | / | / | / |
| Shen et al, 2002 | East Asia | China | FFPE | Conventional PCR | HPV 6/11/16/18 | L1 | 55 ESCC | 36 | / | / | / | / | / |
| Matsha et al, 2002 | Africa | South Africa | FFPE | Conventional PCR | Amy | MY09/11, GP5+/6+ | 50 ESCC | 23 | / | / | / | / | / |
| Li et al, 2002 | East Asia | China | FFPE | Conventional PCR | HPV 16/18 | E6, E7 | 62 | 39 | / | / | / | / | / |
| Hasegawa et al, 2002 | East Asia | Japan | FFPE | Conventional PCR | Any | MY09/11 and type-specific | 48 ESCC | 20 | / | / | / | / | / |
| Sobti et al, 2002 | South Asia | India | FFT | Conventional PCR | Any | GP5+/6+ or CP65/70 | 27 | 20 | / | / | / | / | / |

| | | | | | | | | | | | | | |
|---|---|---|---|---|---|---|---|---|---|---|---|---|---|
| Peixoto Guimaraes et al, 2001 | East Asia | China | FFT | Conventional PCR | Any | GP5+/6+ | 32 ESCC | 2 | 57 | 4 | / | / | / |
| Chen et al, 2001 | East Asia | China | FFPE | Conventional PCR | Any | L1, HPV 16 Specific, HPV 18 Specific | 30 | 19 | / | / | / | / | / |
| Astori et al, 2000 | Europe | Italy | FFT | Conventional PCR | Any | MY09/11 | 14 ESCC | 6 | / | / | / | / | / |
| | | | | | | | 3 EAC | 1 | / | / | / | / | / |
| Tripodi et al, 2000 | East Asia | China | FFPE | Conventional PCR | Amy | MY09/11 | 700 ESCC | 106 | / | / | / | / | / |
| | | | | In Situ Hybridization | Amy | / | 700 ESCC | 116 | / | / | / | / | / |
| Talamini et al, 2000 | Europe | Italy | FFPE | Conventional PCR | Amy | MY09/11, GP5+/6+ | 45 ESCC | 0 | / | / | / | / | / |
| Lambot et al, 2000 | Europe | Belgium | FFPE | Conventional PCR | Any | MY09/11 | 21 ESCC | 1 | / | / | / | / | / |
| Kawaguchi et al, 2000 | East Asia | Japan | FFT | Conventional PCR | HPV 16/18 | HPV 16 Specific, HPV 18 Specific | 75 ESCC | 17 | / | / | / | / | / |
| Kamath et al, 2000 | North America | USA | FFT | Conventional PCR | Any | L1 | 22 ESCC | 0 | / | / | / | / | / |
| | | | | | | | 24 EAC | 1 | / | / | / | / | / |
| Chang et al, 2000 | East Asia | China | FFPE | Conventional PCR | Any | L1 | 103 ESCC | 17 | / | / | / | / | / |
| | | | | In Situ Hybridization | Any | / | 103 ESCC | 6 | / | / | / | / | / |
| Chang et al, 2000 | East Asia | China | FFPE | In Situ Hybridization | HPV 6/11/16/18/30/53 | / | 700 ESCC | 118 | / | / | / | / | / |

Note: FFPE = formalin-fixed paraffin-embedded; FFT: Fresh Frozen Tissue; PCR = polymerase chain reaction; OR = odds ratio; LCI = lower confidence interval; UCI = upper confidence interval; HPV = human papillomavirus; E6/E7 = early region oncogenes E6 and E7 of HPV.

**Supplementary Table S2.** Quality assessment of included studies reporting HPV detection rates using the Newcastle–Ottawa Scale

| Study | Study Design | Selection (Max 5) | Comparability (Max 2) | Outcome Assessment (Max 3) | Overall Score (Max 10) | Overall Risk of Bias |
|---|---|---|---|---|---|---|
| Reynders C, 2025 | Cross-sectional | 4 | 2 | 3 | 9 | Low |
| De Oliveira, 2025 | Nested Case-Control | 3 | 2 | 3 | 8 | Low |
| Qu F, 2024 | Retrospective Cohort | 3 | 1 | 2 | 6 | Moderate |
| Ndemela L M, 2024 | Cross-sectional | 3 | 1 | 2 | 6 | Moderate |
| Kotian S, 2024 | Cross-sectional | 3 | 1 | 2 | 6 | Moderate |
| Yuan W, 2023 | Cross-sectional | 4 | 1 | 3 | 8 | Low |
| Maroga N, 2023 | Cross-sectional | 3 | 1 | 2 | 6 | Moderate |
| Burassakarn A, 2023 | Cross-sectional | 4 | 2 | 3 | 9 | Low |
| Sadeghian Z, 2022 | Cross-sectional | 3 | 2 | 3 | 8 | Low |
| Munari F F, 2022 | Cross-sectional | 4 | 2 | 3 | 9 | Low |
| Zhang Y, 2021 | Cross-sectional | 3 | 1 | 2 | 6 | Moderate |
| Woellner L F A, 2021 | Cross-sectional | 3 | 1 | 2 | 6 | Moderate |
| Ishida H, 2021 | Cross-sectional | 4 | 2 | 3 | 9 | Low |
| Zheng Y, 2020 | Cross-sectional | 4 | 1 | 3 | 8 | Low |
| Zhang C J, 2020 | Cross-sectional | 3 | 1 | 2 | 6 | Moderate |
| Sultana N, 2020 | Cross-sectional | 3 | 1 | 3 | 7 | Moderate |
| Singh T, 2020 | Cross-sectional | 3 | 1 | 2 | 6 | Moderate |
| Kanaan C, 2020 | Cross-sectional | 4 | 2 | 3 | 9 | Low |
| Inoue M, 2020 | Retrospective Study | 4 | 2 | 3 | 9 | Low |
| Dinc B, 2020 | Cross-sectional | 3 | 1 | 2 | 6 | Moderate |
| Tasneem M, 2019 | Cross-sectional | 3 | 1 | 2 | 6 | Moderate |
| Parameshwaran K, 2019 | Cross-sectional | 3 | 1 | 2 | 6 | Moderate |
| Leon ME, 2019 | Case-Control | 4 | 2 | 3 | 9 | Low |

| | | | | | | |
|---|---|---|---|---|---|---|
| Bas Y, 2019 | Cross-sectional | 3 | 1 | 2 | 6 | Moderate |
| VošMik M, 2018 | Cross-sectional | 3 | 1 | 2 | 6 | Moderate |
| Li S, 2018 | Cross-sectional | 4 | 1 | 2 | 7 | Moderate |
| Ghaffar M, 2018 | Cross-sectional | 3 | 1 | 2 | 6 | Moderate |
| Geßner A L, 2018 | Cross-sectional | 4 | 2 | 3 | 9 | Low |
| Cheah P L, 2018 | Cross-sectional | 3 | 1 | 2 | 6 | Moderate |
| Zhang D, 2017 | Cross-sectional | 4 | 2 | 2 | 8 | Low |
| Rajendra S, 2017 | Cross-sectional | 4 | 2 | 3 | 9 | Low |
| Pastrez PRA, 2017 | Cross-sectional | 4 | 1 | 2 | 7 | Moderate |
| da Costa AM, 2017 | Prospective Study | 4 | 2 | 3 | 9 | Low |
| Chan PKS, 2017 | Cross-sectional | 4 | 2 | 2 | 8 | Low |
| Yahyapour Y, 2016 | Cross-sectional | 3 | 1 | 2 | 6 | Moderate |
| Soheili F, 2016 | Cross-sectional | 3 | 1 | 3 | 7 | Moderate |
| Prakash Saxena P U, 2016 | Prospective Study | 4 | 2 | 3 | 9 | Low |
| Antonsson A, 2016 | Cross-sectional | 4 | 1 | 2 | 7 | Moderate |
| Zou N, 2015 | Cross-sectional | 4 | 2 | 3 | 9 | Low |
| Wang W L, 2015 | Cross-sectional | 4 | 2 | 3 | 9 | Low |
| Mehryar M M, 2015 | Cross-sectional | 4 | 1 | 2 | 7 | Moderate |
| Kumar R, 2015 | Retrospective cohort | 3 | 1 | 2 | 6 | Moderate |
| Kayamba V, 2015 | Case-control | 3 | 2 | 3 | 8 | Low |
| Georgantis G, 2015 | Case-control | 3 | 1 | 2 | 6 | Moderate |
| Dong H C, 2015 | Case-control | 3 | 1 | 2 | 6 | Moderate |
| Zhang D H, 2014 | Case-control | 3 | 2 | 2 | 7 | Moderate |
| Yu Q, 2014 | Case-control | 4 | 2 | 3 | 9 | Low |
| Yang J, 2014 | Case-control | 4 | 2 | 3 | 9 | Low |
| Teng H, 2014 | Cross-sectional | 3 | 1 | 2 | 6 | Moderate |

| | | | | | | |
|---|---|---|---|---|---|---|
| Ludmir E B, 2014 | Retrospective Cohort | 3 | 1 | 2 | 6 | Moderate |
| Liu H Y, 2014 | Cross-sectional | 3 | 1 | 2 | 6 | Moderate |
| He Z, 2014 | Case-control | 4 | 2 | 3 | 9 | Low |
| Cui X, 2014 | Case-control | 3 | 1 | 2 | 6 | Moderate |
| Chen W G, 2014 | Case-control | 3 | 1 | 2 | 6 | Moderate |
| Cao F, 2014 | Retrospective Cohort | 3 | 1 | 2 | 6 | Moderate |
| Yang J, 2013 | Case-control | 3 | 2 | 3 | 8 | Low |
| Yahyapour Y, 2013 | Cross-sectional | 3 | 1 | 2 | 6 | Moderate |
| Wang Y F, 2013 | Cross-sectional | 4 | 1 | 2 | 7 | Moderate |
| Vaiphei K, 2013 | Cross-sectional | 3 | 2 | 2 | 7 | Moderate |
| Schäfer G, 2013 | Cross-sectional | 3 | 1 | 2 | 6 | Moderate |
| Rajendra S, 2013 | Cross-sectional | 4 | 2 | 3 | 9 | Low |
| Qi Z, 2013 | Cross-sectional | 3 | 2 | 2 | 7 | Moderate |
| Mohiuddin M K, 2013 | Cross-sectional | 3 | 1 | 2 | 6 | Moderate |
| Liu T, 2013 | Cross-sectional | 3 | 1 | 2 | 6 | Moderate |
| Hu J M, 2013 | Cross-sectional | 4 | 2 | 3 | 9 | Low |
| Hu J, 2013 | Case-control | 3 | 1 | 2 | 6 | Moderate |
| Haeri H, 2013 | Case-control | 3 | 1 | 2 | 6 | Moderate |
| Feng S, 2013 | Cross-sectional | 3 | 1 | 2 | 6 | Moderate |
| Antunes L C M, 2013 | Cross-sectional | 4 | 2 | 3 | 9 | Low |
| Qu P, 2012 | Cross-sectional | 3 | 1 | 2 | 6 | Moderate |
| Noori S, 2012 | Cross-sectional | 3 | 1 | 2 | 6 | Moderate |
| Lofdahl HE, 2012 | Cross-sectional | 4 | 1 | 2 | 7 | Moderate |
| Hu J, 2012 | Case-control | 4 | 1 | 2 | 7 | Moderate |
| Herbster S, 2012 | Cross-sectional | 4 | 1 | 2 | 7 | Moderate |
| Gupta N, 2012 | Cross-sectional | 3 | 1 | 2 | 6 | Moderate |

| | | | | | | |
|---|---|---|---|---|---|---|
| Guo F, 2012 | Case-control | 4 | 2 | 3 | 9 | Low |
| Dąbrowski A, 2012 | Cross-sectional | 4 | 1 | 2 | 7 | Moderate |
| Abdirad A, 2012 | Cross-sectional | 3 | 1 | 2 | 6 | Moderate |
| Zhang Q Y, 2011 | Cross-sectional | 3 | 2 | 3 | 8 | Low |
| Zhang D H, 2011 | Cross-sectional | 4 | 2 | 3 | 9 | Low |
| Malik S M, 2011 | Case-Control | 3 | 2 | 3 | 8 | Low |
| Iyer A, 2011 | Cross-sectional | 4 | 2 | 3 | 9 | Low |
| Hussain S, 2011 | Cross-sectional | 4 | 2 | 3 | 9 | Low |
| Goto A, 2011 | Multi-center cross-sectional | 4 | 1 | 2 | 7 | Moderate |
| Cui M C, 2011 | Cross-sectional | 2 | 0 | 2 | 4 | High |
| Castillo A, 2011 | Cross-sectional | 4 | 2 | 2 | 8 | Low |
| Hasim A, 2011 | Case-control | 4 | 2 | 3 | 9 | Low |
| Zhang D, 2010 | Case-control within cross-sectional | 3 | 1 | 2 | 6 | Moderate |
| Wang X, 2010 | Cross-sectional | 4 | 2 | 2 | 8 | Low |
| Liu W K, 2010 | Single-center cross-sectional | 3 | 1 | 2 | 6 | Moderate |
| Koshiol J, 2010 | Cross-sectional | 4 | 1 | 3 | 8 | Low |
| Antonsson A, 2010 | Cross-sectional | 4 | 1 | 2 | 7 | Moderate |
| Zhao X Y, 2009 | Cross-sectional | 3 | 1 | 2 | 6 | Moderate |
| Tornesello M L, 2009 | Cross-sectional | 3 | 0 | 2 | 5 | Moderate |
| Herrera-Goepfert R, 2009 | Cross-sectional | 3 | 0 | 2 | 5 | Moderate |
| Liao P H, 2009 | Cross-sectional | 3 | 1 | 2 | 6 | Moderate |
| Bellizzi A M, 2009 | Cross-sectional | 3 | 1 | 3 | 7 | Moderate |
| Yang W, 2008 | Cross-sectional | 3 | 1 | 3 | 7 | Moderate |

| | | | | | | |
|---|---|---|---|---|---|---|
| Lyronis I D, 2008 | Cross-sectional | 3 | 1 | 3 | 7 | Moderate |
| Lu X M, 2008 | Cross-sectional | 3 | 1 | 3 | 7 | Moderate |
| Li S Y, 2008 | Cross-sectional | 2 | 0 | 2 | 4 | High |
| Koh J S, 2008 | Cross-sectional | 3 | 1 | 2 | 6 | Moderate |
| Bognar G, 2008 | Cross-sectional | 3 | 1 | 2 | 6 | Moderate |
| Zhou Y, 2007 | Cross-sectional | 3 | 1 | 3 | 7 | Moderate |
| Shuyama K, 2007 | Cross-sectional | 3 | 1 | 2 | 6 | Moderate |
| Pantelis A, 2007 | Cross-sectional | 3 | 1 | 2 | 6 | Moderate |
| Mir M M, 2007 | Cross-sectional | 3 | 1 | 2 | 6 | Moderate |
| Matsha T, 2007 | Cross-sectional | 3 | 1 | 2 | 6 | Moderate |
| Liu M, 2007 | Cross-sectional | 3 | 1 | 2 | 6 | Moderate |
| Far A E, 2007 | Cross-sectional | 3 | 1 | 2 | 6 | Moderate |
| Dai M, 2007 | Cross-sectional | 4 | 1 | 3 | 8 | Low |
| Yao P F, 2006 | Cross-sectional | 3 | 1 | 2 | 6 | Moderate |
| Qi Z L, 2006 | Case-Control | 3 | 1 | 3 | 7 | Moderate |
| Damin A P S, 2006 | Cross-sectional | 4 | 1 | 2 | 7 | Moderate |
| Kamangar F, 2006 | Cross-sectional | 5 | 2 | 3 | 10 | Low |
| Dreilich M, 2006 | Cross-sectional | 3 | 1 | 2 | 6 | Moderate |
| Castillo A, 2006 | Cross-sectional | 3 | 1 | 2 | 6 | Moderate |
| Zhu L Z, 2005 | Cross-sectional | 3 | 1 | 2 | 6 | Moderate |
| White R E, 2005 | Cross-sectional | 3 | 1 | 2 | 6 | Moderate |
| Lyronis I D, 2005 | Cross-sectional | 3 | 1 | 2 | 6 | Moderate |
| Liu W K, 2005 | Cross-sectional | 3 | 1 | 2 | 6 | Moderate |
| Katiyar S, 2005 | Cross-sectional | 3 | 1 | 2 | 6 | Moderate |
| Farhadi M, 2005 | Cross-sectional | 3 | 1 | 2 | 6 | Moderate |
| Cao B, 2005 | Case-Control | 4 | 2 | 3 | 9 | Low |

| | | | | | | |
|---|---|---|---|---|---|---|
| Bahnassy A A, 2005 | Cross-sectional | 4 | 1 | 2 | 7 | Moderate |
| Xu C L, 2004 | Cross-sectional | 3 | 1 | 2 | 6 | Moderate |
| Si H X, 2004 | Cross-sectional | 3 | 1 | 2 | 6 | Moderate |
| Lu X M, 2004 | Cross-sectional | 3 | 1 | 2 | 6 | Moderate |
| Acevedo-Nuño, 2004 | Cross-sectional | 3 | 1 | 2 | 6 | Moderate |
| Zhou X B, 2003 | Cross-sectional | 3 | 1 | 2 | 6 | Moderate |
| Weston A C, 2003 | Cross-sectional | 3 | 1 | 2 | 6 | Moderate |
| Van Doornum GJJ, 2003 | Cross-sectional | 3 | 1 | 2 | 6 | Moderate |
| Si H X, 2003 | Cross-sectional | 3 | 1 | 3 | 7 | Moderate |
| Liu YL, 200 | Cross-sectional | 3 | 1 | 2 | 6 | Moderate |
| Awerkiew S, 2003 | Cross-sectional | 3 | 1 | 2 | 6 | Moderate |
| Xu W G, 2003 | Cross-sectional | 4 | 1 | 3 | 8 | Low |
| Shen ZY, 2002 | Case-Control | 4 | 2 | 3 | 9 | Low |
| Matsha T, 2002 | Cross-sectional | 3 | 2 | 3 | 8 | Low |
| Li T, 2002 | Case-Control | 4 | 2 | 2 | 8 | Low |
| Hasegawa M, 2002 | Cross-sectional | 3 | 1 | 3 | 7 | Moderate |
| Sobti R C, 2001 | Cross-sectional | 3 | 1 | 2 | 6 | Moderate |
| Guimaraes D P, 2001 | Cross-sectional | 3 | 2 | 3 | 8 | Low |
| Lu Z, 2001 | Cross-sectional | 3 | 1 | 2 | 6 | Moderate |
| Astori G, 2001 | Cross-sectional | 4 | 1 | 2 | 7 | Moderate |
| Tripodi S, 2000 | Cross-sectional | 4 | 1 | 2 | 7 | Moderate |
| Talamini G, 2000 | Cross-sectional | 3 | 1 | 2 | 6 | Moderate |
| Lambot M A, 2000 | Cross-sectional | 3 | 1 | 2 | 6 | Moderate |
| Kamath A M, 2000 | Cross-sectional | 4 | 1 | 2 | 7 | Moderate |
| Chang F, 2000 | Cross-sectional | 3 | 1 | 2 | 6 | Moderate |

**Supplementary Table S3**. Quality assessment of included case–control studies reporting the association between HPV infection and esophageal cancer using the Newcastle–Ottawa Scale

| Author | Year | Selection | | | | Comparability | | Exposure | | | Score |
|---|---|---|---|---|---|---|---|---|---|---|---|
| | | Adequacy of the case definition | Representativeness of cases | Choice of controls | Definition of control | Study controls for age | Study controls for any additional important factor | Exposure assessment | The method of exposure assessment | Non-response rate | |
| Burassakarn et al | 2023 | ★ | ★ | ★ | ★ | | | ★ | ★ | | 6 |
| Parameshwaran et al | 2019 | ★ | ★ | ★ | ★ | | | ★ | ★ | | 6 |
| Yahyapour et al | 2016 | ★ | ★ | ★ | ★ | | | ★ | ★ | | 6 |
| Zou et al | 2015 | ★ | ★ | ★ | ★ | | | ★ | ★ | | 6 |
| Kayamba et al | 2015 | ★ | ★ | ★ | ★ | ★ | ★ | ★ | ★ | | 8 |
| Dong et al | 2015 | ★ | ★ | ★ | ★ | | | ★ | ★ | | 6 |
| Yu et al | 2014 | ★ | ★ | ★ | ★ | ★ | ★ | ★ | ★ | | 8 |
| Yang et al | 2014 | ★ | ★ | ★ | ★ | | | ★ | ★ | | 6 |
| Liu et al | 2014 | ★ | ★ | ★ | ★ | | | ★ | ★ | | 6 |
| He et al | 2014 | ★ | ★ | ★ | ★ | | | ★ | ★ | | 6 |
| Cui et al | 2014 | ★ | ★ | ★ | ★ | | | ★ | ★ | | 6 |
| Qi et al | 2013 | ★ | ★ | ★ | ★ | | | ★ | ★ | | 6 |
| Hu et al | 2013 | ★ | ★ | ★ | ★ | | | ★ | ★ | | 6 |
| Hu et al | 2012 | ★ | ★ | ★ | ★ | | | ★ | ★ | | 6 |
| Guo et al | 2012 | ★ | ★ | ★ | ★ | ★ | ★ | ★ | ★ | | 8 |
| Zhang et al | 2011 | ★ | ★ | ★ | ★ | | | ★ | ★ | | 6 |
| Lyronis et al | 2008 | ★ | ★ | ★ | ★ | | | ★ | ★ | | 6 |

Case-control Studies: **Selection** ① Adequacy of the case definition★ ② Representativeness of cases★ ③ Choice of controls★ ④ Definition of control★. **Comparability** ① Comparability of case-controls on the basis of the design or analysis★★. **Exposure** ① Exposure assessment★ ② The method of exposure assessment★ ③ Non-response rate★.

**Supplementary Table S4.** PCR-based detection rates of HPV stratified by primer type

| **Characteristic** | **Studies (*N*)** | **EC patients (*N*)** | **HPV positive (*N*)** | **Prevalence (*95% CI*)** | **Heterogeneity (Random Effects Model)** | |
|---|---|---|---|---|---|---|
| | | | | | $I^2$ | *P* value |
| MY09/11 | 14 | 1877 | 593 | 0.34 (0.22, 0.48) | 97.2% | < 0.0001 |
| GP5+/6+ | 24 | 3681 | 1414 | 0.35 (0.21, 0.51) | 98.5% | < 0.0001 |
| SPF10 | 4 | 477 | 314 | 0.49 (0, 1.00) | 99.4% | < 0.0001 |
| L1 | 12 | 883 | 254 | 0.30 (0.08, 0.58) | 98.0% | < 0.0001 |
| E7 | 12 | 1344 | 664 | 0.41 (0.23, 0.60) | 97.8% | < 0.0001 |
| GP5+/6+ combined with MY09/11 | 21 | 1970 | 454 | 0.27 (0.14, 0.42) | 95.7% | < 0.0001 |
| GP5+/6+ combined with SPF10 | 2 | 116 | 19 | 0.10 (0, 1.00) | 97.1% | < 0.0001 |
| GP5+/6+ combined with E7 | 1 | 316 | 204 | 0.65 (0.59, 0.70) | / | / |
| Type specific | 10 | 973 | 333 | 0.31 (0.24, 0.39) | 68.8% | 0.0007 |

**Supplementary Table S5.** HPV genotype–specific prevalence stratified by detection method and histological subtype

| Characteristic | Studies (*N*) | EC patients (*N*) | HPV positive (*N*) | Prevalence (*95% CI*) | Heterogeneity (Random Effects Model) | |
|---|---|---|---|---|---|---|
| | | | | | $I^2$ | *P* value |
| **Polymerase Chain Reaction** | | | | | | |
| **HPV-16** | | | | | | |
| ESCC | 56 | 7718 | 2152 | 0.24 (0.18, 0.30) | 96.4% | <0.0001 |
| EAC | 3 | 223 | 39 | 0.15 (0.02, 0.34) | 52.0% | 0.1243 |
| **HPV-18** | | | | | | |
| ESCC | 32 | 5203 | 736 | 0.09 (0.06, 0.14) | 96.2% | <0.0001 |
| EAC | 2 | 187 | 22 | 0.11 (0.00, 0.68) | 27.4% | 0.2406 |
| **In Situ Hybridization** | | | | | | |
| **HPV-16** | | | | | | |
| ESCC | 7 | 1293 | 272 | 0.34 (0.08, 0.65) | 98.9% | <0.0001 |
| **HPV-18** | | | | | | |
| ESCC | 2 | 803 | 14 | 0.02 (0.00, 0.05) | 0.0% | 0.6966 |
| **Immunohistochemistry** | | | | | | |
| **HPV-16** | | | | | | |
| ESCC | 6 | 309 | 124 | 0.46 (0.20, 0.73) | 90.0% | <0.0001 |
| **Enzyme-Linked Immunosorbent Assay** | | | | | | |
| **HPV-16** | | | | | | |
| ESCC | 7 | 2818 | 254 | 0.30 (0.12, 0.51) | 98.9% | <0.0001 |
| EAC | 1 | 48 | 11 | 0.23 (0.12, 0.37) | / | / |
| **HPV-18** | | | | | | |
| ESCC | 2 | 1534 | 83 | 0.06 (0.00, 0.30) | 35.2% | 0.2143 |

| | | | | | | |
|---|---|---|---|---|---|---|
| **Other** | | | | | | |
| **HPV-16** | | | | | | |
| ESCC | 4 | 416 | 116 | 0.31 (0.00, 0.86) | 98.5% | <0.0001 |
| EAC | 1 | 28 | 4 | 0.14 (0.04, 0.33) | / | / |
| **HPV-18** | | | | | | |
| ESCC | 2 | 150 | 16 | 0.10 (0.00, 0.95) | 73.6% | 0.0517 |
| EAC | 1 | 28 | 5 | 0.18 (0.06, 0.37) | / | / |

**Supplementary Figure S1.** Prevalence of biologically active HPV infection in esophageal cancer

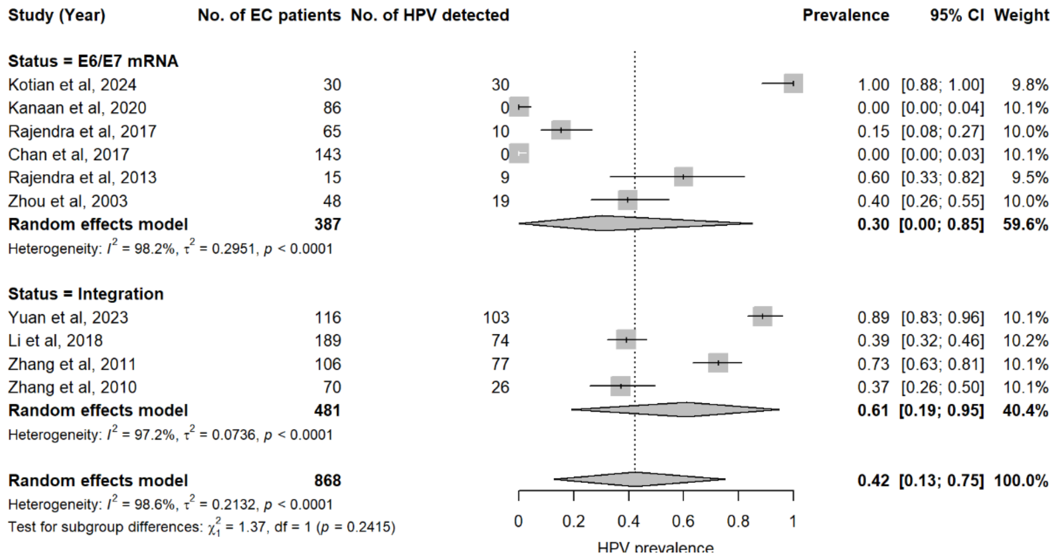

**Supplementary Figure S2.** Global distribution of pooled HPV prevalence in esophageal cancer based on PCR studies. Pooled prevalence estimates are derived from the meta-analysis.

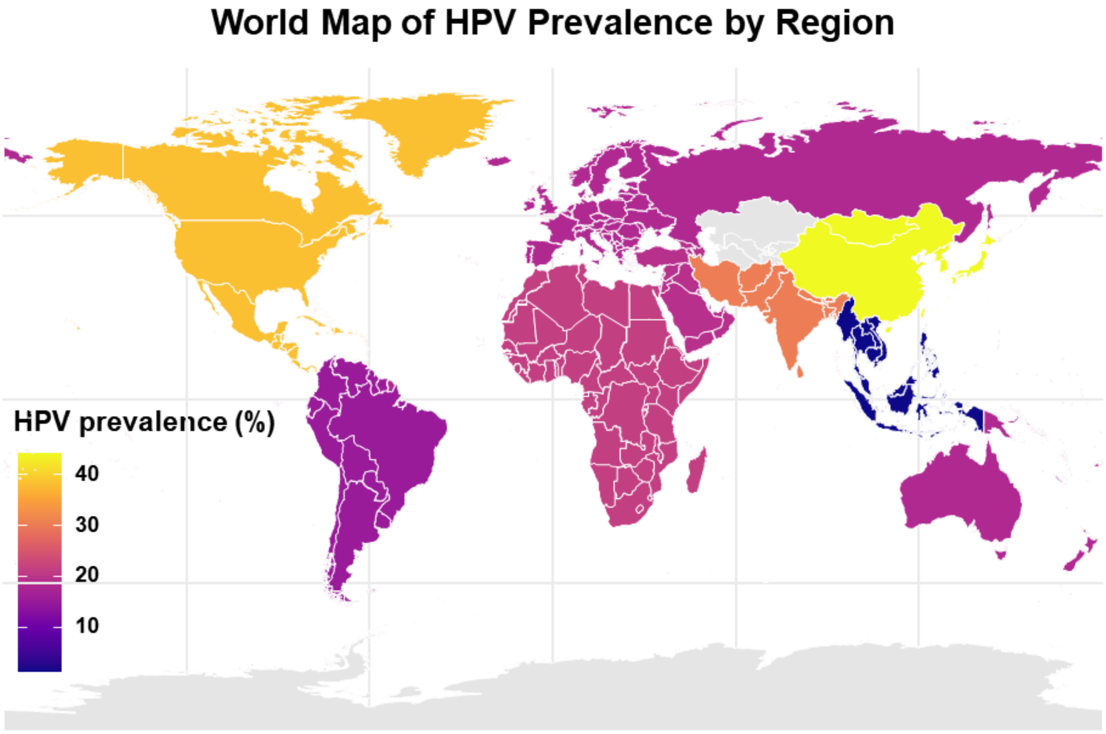

**Supplementary Figure S3.** Forest plot of the overall association between HPV infection and esophageal squamous cell carcinoma (ESCC), by detection method. Each horizontal line represents a study-specific OR with 95% CI for the association between HPV infection and ESCC. The pooled OR was calculated using a random-effects model. Study weights are proportional to inverse-variance and indicated on the right.

| Study (Year) | Case (N) | HPV (+) | Control (N) | HPV (+) | OR | 95%-CI | Weight (common) | Weight (random) |
|---|---|---|---|---|---|---|---|---|
| **Detection_method = PCR** | | | | | | | | |
| Cui et al, 2014 | 183 | 58 | 89 | 8 | 4.70 | [2.13; 10.37] | 2.4% | 5.1% |
| Guo et al, 2012 | 300 | 93 | 900 | 61 | 6.40 | [4.43; 9.25] | 11.2% | 7.2% |
| Hu et al, 2012 | 200 | 82 | 150 | 24 | 3.62 | [2.15; 6.09] | 5.6% | 6.5% |
| Hu et al, 2013 | 150 | 55 | 150 | 24 | 3.04 | [1.76; 5.26] | 5.1% | 6.4% |
| Kayamba et al, 2015 | 44 | 2 | 48 | 1 | 2.20 | [0.11; 44.54] | 0.2% | 0.9% |
| Lyronis et al, 2008 | 30 | 17 | 32 | 6 | 5.66 | [1.80; 17.79] | 1.2% | 3.6% |
| Yahyapour et al, 2016 | 51 | 16 | 45 | 20 | 2.45 | [0.99; 6.05] | 1.9% | 4.6% |
| Zou et al, 2015 | 316 | 204 | 66 | 12 | 8.20 | [4.24; 15.83] | 3.5% | 5.8% |
| **Common effect model** | | | | | **4.79** | **[3.84; 5.98]** | **31.1%** | **.** |
| **Random effects model** | | | | | **4.57** | **[3.31; 6.32]** | **.** | **40.1%** |
| Heterogeneity: $I^2 = 37.2\%$, $\tau^2 = 0.0821$, $p = 0.1327$ | | | | | | | | |
| **Detection_method = ELISA** | | | | | | | | |
| He et al, 2014 | 1435 | 178 | 2071 | 73 | 3.60 | [2.55; 5.09] | 12.7% | 7.3% |
| He et al, 2014 | 1435 | 75 | 2071 | 55 | 1.10 | [0.71; 1.71] | 7.8% | 6.9% |
| Kamangar et al, 2006 | 99 | 12 | 381 | 29 | 1.70 | [0.76; 3.80] | 2.4% | 5.1% |
| Kamangar et al, 2006 | 99 | 8 | 381 | 35 | 0.90 | [0.30; 2.70] | 1.3% | 3.8% |
| Qi et al, 2013 | 225 | 87 | 224 | 65 | 1.64 | [1.07; 2.52] | 8.3% | 7.0% |
| Yang et al, 2014 | 313 | 170 | 314 | 136 | 1.56 | [1.14; 2.13] | 15.6% | 7.5% |
| Yu et al , 2014 | 308 | 168 | 309 | 134 | 1.74 | [1.25; 2.43] | 13.8% | 7.4% |
| **Common effect model** | | | | | **1.81** | **[1.55; 2.12]** | **61.9%** | **.** |
| **Random effects model** | | | | | **1.71** | **[1.23; 2.37]** | **.** | **44.9%** |
| Heterogeneity: $I^2 = 73.5\%$, $\tau^2 = 0.1278$, $p = 0.0009$ | | | | | | | | |
| **Detection_method = Other** | | | | | | | | |
| Burassakarn et al, 2023 | 72 | 32 | 105 | 23 | 3.20 | [1.42; 7.21] | 2.3% | 5.0% |
| Dong et al, 2015 | 89 | 46 | 49 | 14 | 2.67 | [1.27; 5.63] | 2.8% | 5.4% |
| Liu et al, 2014 | 78 | 54 | 30 | 10 | 4.50 | [1.83; 11.06] | 1.9% | 4.6% |
| **Common effect model** | | | | | **3.27** | **[2.05; 5.22]** | **7.0%** | **.** |
| **Random effects model** | | | | | **3.27** | **[2.05; 5.22]** | **.** | **15.0%** |
| Heterogeneity: $I^2 = 0\%$, $\tau^2 = 0$, $p = 0.6800$ | | | | | | | | |
| **Common effect model** | | | | | **2.56** | **[2.26; 2.89]** | **100.0%** | **.** |
| **Random effects model** | | | | | **2.75** | **[2.05; 3.70]** | **.** | **100.0%** |

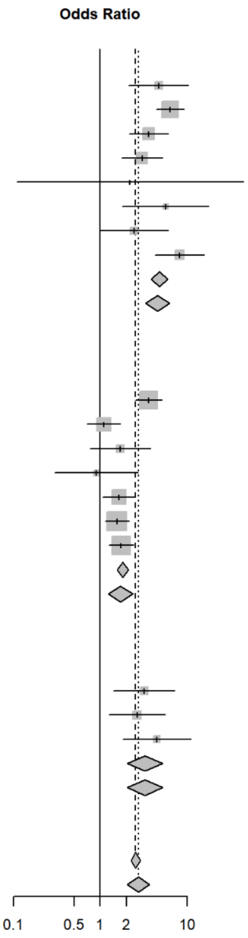


Heterogeneity: $I^2 = 80.0\%$, $\tau^2 = 0.2760$, $p < 0.0001$

Test for subgroup differences (common effect): $\chi^2_2 = 50.27$, df = 2 ($p < 0.0001$)

Test for subgroup differences (random effects): $\chi^2_2 = 17.89$, df = 2 ($p = 0.0001$)

**Supplementary Figure S4.** Forest plot of the overall association between HPV infection and esophageal adenocarcinoma (EAC), by detection method. Each horizontal line represents a study-specific OR with 95% CI for the association between HPV infection and EAC. The pooled OR was calculated using a random-effects model. Study weights are proportional to inverse-variance and indicated on the right.

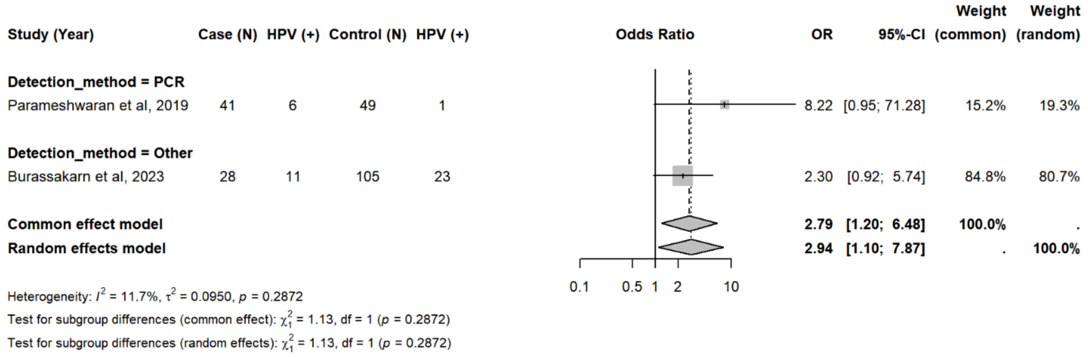

**Supplementary Figure S5.** Forest plot of the overall association between HPV 16 infection and esophageal cancer, by detection method. Each horizontal line represents a study-specific OR with 95% CI for the association between HPV 16 infection and esophageal cancer. The pooled OR was calculated using a random-effects model. Study weights are proportional to inverse-variance and indicated on the right.

| Study (Year) | Case (N) | HPV (+) | Control (N) | HPV (+) | OR | 95%-CI | Weight (common) | Weight (random) |
|---|---|---|---|---|---|---|---|---|
| **Detection_method = PCR** | | | | | | | | |
| Guo et al, 2012 | 300 | 70 | 900 | 21 | 12.80 | [7.58; 21.63] | 7.4% | 9.4% |
| Hu et al, 2012 | 200 | 82 | 150 | 24 | 3.62 | [2.15; 6.09] | 7.5% | 9.4% |
| Hu et al, 2013 | 150 | 55 | 150 | 24 | 3.04 | [1.76; 5.26] | 6.8% | 9.2% |
| Zhang et al, 2011 | 70 | 28 | 100 | 8 | 7.70 | [3.23; 18.36] | 2.7% | 7.3% |
| Zou et al, 2015 | 316 | 130 | 66 | 10 | 3.94 | [1.94; 8.01] | 4.1% | 8.3% |
| **Common effect model** | | | | | **5.25** | **[4.01; 6.85]** | **28.5%** | . |
| **Random effects model** | | | | | **5.27** | **[3.02; 9.21]** | . | **43.6%** |
| Heterogeneity: $I^2 = 78.1\%$, $\tau^2 = 0.3007$, $p = 0.0011$ | | | | | | | | |
| **Detection_method = ELISA** | | | | | | | | |
| Guo et al, 2012 | 300 | 51 | 900 | 28 | 6.10 | [3.75; 9.91] | 8.6% | 9.6% |
| He et al, 2014 | 1435 | 178 | 2071 | 73 | 3.60 | [2.55; 5.09] | 17.0% | 10.3% |
| Kamangar et al, 2006 | 99 | 12 | 381 | 29 | 1.70 | [0.76; 3.80] | 3.1% | 7.7% |
| Yang et al, 2014 | 313 | 170 | 314 | 136 | 1.56 | [1.14; 2.13] | 20.9% | 10.5% |
| Yu et al , 2014 | 308 | 168 | 309 | 134 | 1.74 | [1.25; 2.43] | 18.5% | 10.4% |
| **Common effect model** | | | | | **2.36** | **[1.99; 2.81]** | **68.1%** | . |
| **Random effects model** | | | | | **2.53** | **[1.50; 4.29]** | . | **48.6%** |
| Heterogeneity: $I^2 = 87.1\%$, $\tau^2 = 0.3033$, $p < 0.0001$ | | | | | | | | |
| **Detection_method = Other** | | | | | | | | |
| Dong et al, 2015 | 89 | 44 | 49 | 12 | 3.02 | [1.39; 6.55] | 3.4% | 7.9% |
| **Common effect model** | | | | | **2.99** | **[2.59; 3.45]** | **100.0%** | . |
| **Random effects model** | | | | | **3.54** | **[2.38; 5.25]** | . | **100.0%** |

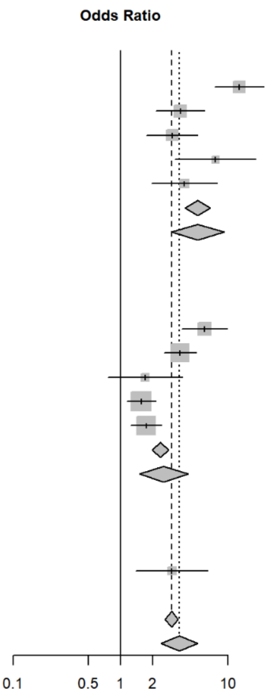


Heterogeneity: $I^2 = 86.4\%$, $\tau^2 = 0.3624$, $p < 0.0001$
Test for subgroup differences (common effect): $\chi^2_2 = 24.05$, df = 2 ($p < 0.0001$)
Test for subgroup differences (random effects): $\chi^2_2 = 3.66$, df = 2 ($p = 0.1606$)

**Supplementary Figure S6.** Forest plot of the overall association between HPV 18 infection and esophageal cancer, by detection method. Each horizontal line represents a study-specific OR with 95% CI for the association between HPV 18 infection and esophageal cancer. The pooled OR was calculated using a random-effects model. Study weights are proportional to inverse-variance and indicated on the right.

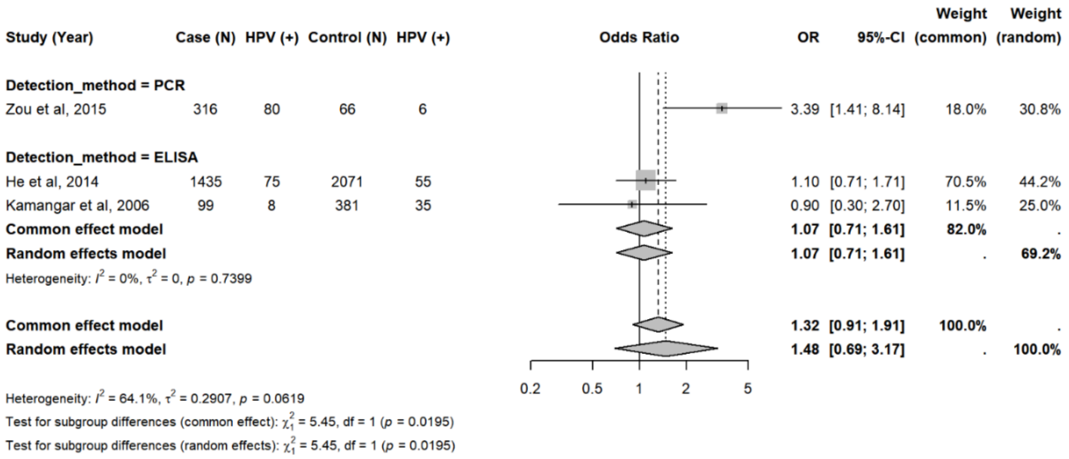

**Supplementary Figure S7.** Forest plot showing the association between human papillomavirus (HPV) infection and esophageal squamous cell carcinoma in sensitivity analyses restricted to high-quality studies (Newcastle–Ottawa Scale score >7)

| Study (Year) | Case (N) | HPV (+) | Control (N) | HPV (+) | OR | 95%-CI | Weight (common) | Weight (random) |
|---|---|---|---|---|---|---|---|---|
| **Detection_method = PCR** | | | | | | | | |
| Guo et al, 2012 | 300 | 93 | 900 | 61 | 6.40 | [4.43; 9.25] | 44.5% | 44.8% |
| Kayamba et al, 2015 | 44 | 2 | 48 | 1 | 2.20 | [0.11; 44.54] | 0.7% | 10.0% |
| **Common effect model** | | | | | **6.30** | **[4.37; 9.08]** | **45.2%** | . |
| **Random effects model** | | | | | **6.30** | **[4.37; 9.08]** | . | **54.8%** |
| Heterogeneity: $I^2 = 0\%$, $\tau^2 = 0$, $p = 0.4898$ | | | | | | | | |
| **Detection_method = ELISA** | | | | | | | | |
| Yu et al , 2014 | 308 | 168 | 309 | 134 | 1.74 | [1.25; 2.43] | 54.8% | 45.2% |
| **Common effect model** | | | | | **3.11** | **[2.43; 3.98]** | **100.0%** | . |
| **Random effects model** | | | | | **3.19** | **[1.10; 9.29]** | . | **100.0%** |

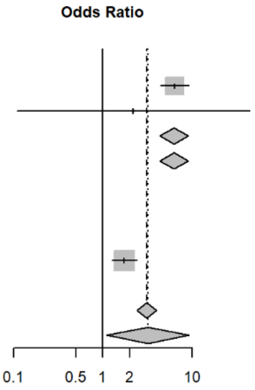


Heterogeneity: $I^2 = 92.4\%$, $\tau^2 = 0.6283$, $p < 0.0001$

Test for subgroup differences (common effect): $\chi^2_1 = 26.01$, df = 1 ($p < 0.0001$)

Test for subgroup differences (random effects): $\chi^2_1 = 26.01$, df = 1 ($p < 0.0001$)
